# Neural sampling from cognitive maps enables goal-directed imagination and planning

**DOI:** 10.1038/s42256-026-01254-4

**Published:** 2026-07-21

**Authors:** Hui Lin, Yukun Yang, Rong Zhao, Giovanni Pezzulo, Wolfgang Maass

**Affiliations:** 1 Department of Precision Instruments, Center for Brain-Inspired Computing Research (CBICR), Tsinghua University, Beijing, China; 2 Institute of Machine Learning and Neural Computation, Graz University of Technology, Graz, Austria; 3 Institute of Cognitive Sciences and Technologies, National Research Council, Rome, Italy

**Keywords:** Computational science, Learning algorithms, Network models

## Abstract

Artificial intelligence systems are becoming more intelligent, but at a very high cost in terms of energy consumption and training requirements. By contrast, our brains only require 20 W of energy, they learn online and they can instantly adjust to changing contingencies. This begs the question what data structures, algorithms and learning methods enable brains to achieve that, and whether these can be ported into artificial devices. We are addressing this question for a core feature of intelligence: the capacity to plan and solve problems, including new problems that involve states that were never encountered before. Here we examine three tools that brains are likely to use for achieving that: cognitive maps, stochastic computing and compositional coding. We integrate these tools into a transparent neural network model, and demonstrate its power for flexible planning and problem-solving. Importantly, this approach is suitable for implementation by in-memory computing and other energy-efficient neuromorphic hardware. In particular, it only requires self-supervised local synaptic plasticity that is suited for on-chip learning. Hence, a core feature of brain intelligence—the capacity to generate solutions to problems that were never encountered before—does not require deep neural networks or large language models, and can be implemented in energy-efficient edge devices.

## Abstract (web summary)

Lin et al. introduce a brain-inspired generative model that provides two key features of intelligence: planning and problem-solving. It uses cognitive maps, stochastic computing and compositional coding, and requires only local synaptic plasticity.

## Main

Planning and problem-solving are at the core of human intelligence. This capability is apparent in multiple domains, such as when planning routes to reach a goal location in a physical environment or when imagining solutions to abstract problems. Importantly, we can apply it also for coping with new challenges that go beyond previous experience.

Numerous experimental data suggest that cognitive maps, that is, data structure for coherently organizing learned experiences by encoding relational information between states and actions, are essential tools that the brain uses for planning. The most widely studied ones are allocentric spatial maps in the rodent hippocampal formation and entorhinal cortex that relate locations in an environment with changes caused by ego-motion^1–5^. They rely crucially on the largely innate grid-cell system for their formation^2,6–8^. These cognitive maps support goal-oriented spatial navigation by providing self-localization and a sense of direction towards goals^9–11^. Recent experimental work in neuroscience and cognitive science goes beyond a purely spatial definition of cognitive maps, suggesting that they are also used by the brain, especially the human brain, for problem-solving in non-spatial domains, including abstract concept spaces^2,12–17^.

Multiple lines of evidence suggest that cognitive maps of the brain not only encode past experience but also serve a prospective function, commonly referred to as imagination, which enables individuals to anticipate and prepare for situations they have never directly experienced. A rodent study showed that sampling from cognitive maps during replay of sequences of place cells in the hippocampus can be decoded as imagined trajectories in two-dimensional (2D) spaces that resemble locomotion plans^18–20^. Furthermore, when novel barriers are introduced in the environment, replay trajectories depict goal-directed trajectories around them^21^.

The human brain is able to imagine and plan also in compositional domains, such as natural language, where a virtually unlimited repertoire of new states and potential goals can be described through new combinations of known components. The underlying brain processes have recently been analysed in ref. ^22^ for the composition of novel 2D silhouettes from a set of building blocks (BBs; tiles), or more precisely, for the NP-hard task to decide whether a given silhouette can be decomposed into these BBs.

We integrate these three identified tools that brains use for planning and problem-solving, cognitive maps, sampling and compositional computing, into a model for planning and problem-solving. This model can be implemented through simple and transparent neural networks that do not require backpropagation or backpropagation through time for learning. Instead, it learns autonomously in an online manner from own experiences, using only biologically plausible rules for local synaptic plasticity that are easy to implement in neuromorphic hardware. We examine the functional capabilities of this model in three very different application domains.

Reinforcement learning (RL) is the standard tool in artificial intelligence (AI) for learning to solve planning or more general problem-solving tasks^23^. However, most RL methods require relearning and substantial computational overhead when the goal changes, and often also deep learning. By contrast, our model can instantly adapt to changes in goals and contingencies through local synaptic plasticity.

Surprisingly, cognitive maps provide a sense of direction even in complex compositional task domains such as composing and decomposing silhouettes from given BBs. Essential for that is that the standard forward model that defines the relational structure of a cognitive map is complemented by an inverse model that maps state difference to neural codes for actions that cause these state differences. Furthermore, very simple inverse models for cognitive maps often suffice, which provide especially attractive generalization capabilities. In general, inverse models have become a standard modelling tool for biological motor control and in robotics^24,25^, but they have apparently not yet been considered in the context of cognitive maps.

We will first demonstrate the functional abilities of our model in 2D spatial navigation. We use a simple model for learning cognitive maps that is based on the grid-cell system, which uses path integration to update the spatial location that is encoded by grid-cell firing^7,8^. To address goal-directed sampling in non-spatial domains, we use a simple model for learning cognitive maps that is based on the learning predictions of action outcomes through local and biologically plausible synaptic plasticity rules^26^. We introduce a generative variant of this cognitive map learner (CML) model, the generative cognitive map learner (GCML), in which observations of next states that result from own actions are replaced by internally generated state predictions. If noise is present in the selection of virtual next actions, one arrives at a probabilistic generative model that permits the sampling of possible trajectories to any given goal in the cognitive map. Importantly, we show that cognitive maps provide a sense of direction even in complex compositional task domains such as composing and decomposing silhouettes from given BBs.

## Results

### Sampling from a 2D cognitive map reproduces brain data on imagined spatial trajectories

Numerous neural recordings during self-generated imagination processes in the brain are available from the rodent hippocampus. In particular, ref. ^18^ has shown that before navigation towards a known goal location (‘home’), the hippocampus produced during specific resting phases (short wave ripples), sequential activity of place cells that could be decoded as imagined trajectories from the current location to the goal location. These decoded trajectories exhibited a startling diversity, even for the same starting and goal location, and were rarely straight (Fig. 2 and supplementary figs. 11 and 17 of ref. ^18^). A subsequent study showed that these decoded trajectories can flexibly reach goals even when this requires rerouting around novel barriers^21^. These findings cannot be easily accounted for by standard sampling approaches used in statistics and machine learning, such as Markov chain Monte Carlo and Monte Carlo tree search^27–29^, since these are lacking the goal-directed feature. Hence, new model for goal-directed stochastic sampling is needed, which we will present here.

**Fig. 1:**
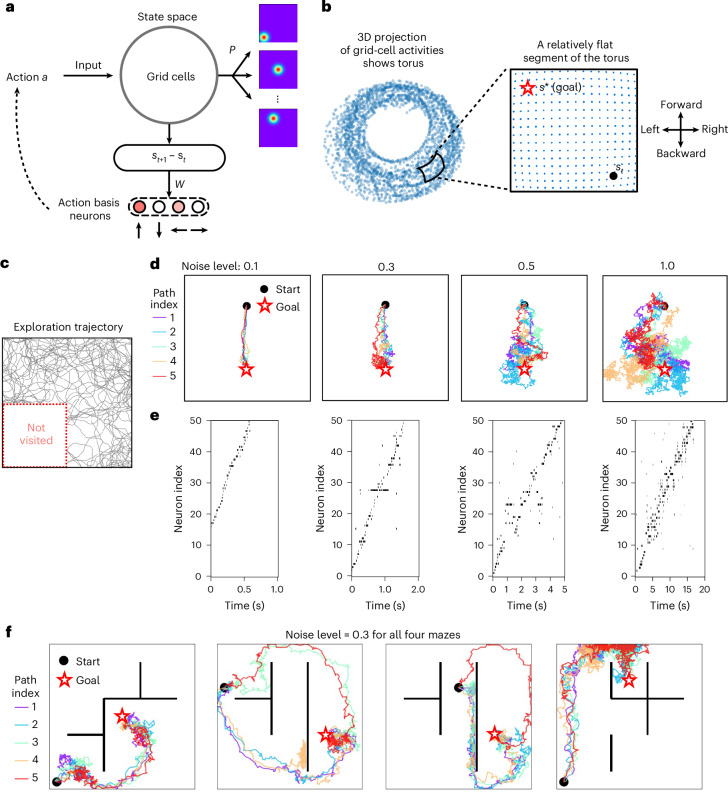
Sampling from a 2D cognitive map supports goal-directed imagination of spatial trajectories. **a**, Model architecture. A standard path integration model for grid cells^53,54^ is used to update grid-cell firing based on movement commands (actions) *a*. These are represented here as (normalized) superpositions of basic movement commands in four directions. Grid cells provide synaptic inputs to an array of place cells that are trained through a simple local learning mechanism. Simultaneously, also synaptic connections **W** from grid cells to basis action neurons are learned by a simple local synaptic plasticity rule, yielding a linear inverse model. **b**, Output of grid cells (for a specific scale) maps a 2D spatial environment onto a segment of the surface of a torus^32^. If the grid cells operate on a sufficiently large scale, this segment is approximately flat for a given 2D arena, thereby supporting navigation based on a sense of direction in two dimensions. **c**, Exploration trajectory used to learn the inverse model **W**. We deliberately left a quarter of the space unvisited, where the trajectory is reflected at its perimeter, to test whether our model can generate imagined trajectories that also visit unexplored parts of the 2D arena. **d**, Impact of different noise levels (0.1, 0.3, 0.5 and 1.0) during action generation on the variance of the generated trajectories. We show five trajectories for each noise level. **e**, Spike trains of 50 selected place cells for one of the trajectories (marked in red) in each corresponding plot of **d**. **f**, Goal-directed trajectories generated by the same model in which repulsive forces from obstacles affect action selection. These trajectories look remarkably similar to those observed in the rodent brain^21^.

We start with a standard model for a cognitive map for spatial navigation that is based on the grid-cell system (Fig. 1a and Methods). Grid cells produce an allocentric map of a 2D environment based on path integration, thereby relating ego-motion to estimates of spatial locations in the 2D environment^2,6–8,30^. Experimental data suggest that the grid-cell system is largely genetically programmed in the rodent, but its development is sped up through spatial navigation^31^. Importantly, for our context, ref. ^20^ shows that the grid-cell system is instrumental in generating imagined trajectories (forward sweeps) in the absence of movements. In fact, their data suggest the presence of a forward model in which grid-cell states are sequentially updated in the direction of an imagined movement. The allocentric map formed by the grid-cell system corresponds on a large scale to a torus^32^. We focus on a local segment of the torus, which is approximately flat (Fig. 1b). Although each grid cell fires for numerous locations in two dimensions, they can be used as basis for training place cells that each fire only around a specific location (Fig. 1a).

Previous analyses and models suggested that the diversity of 2D trajectories that are decoded from the sequential activity of place cells exhibits signs of an underlying stochastic sampling process^33,34^. However, a model was missing that could reproduce the sampling of goal-directed trajectories, such as the virtual trajectories to a home location in the rodent^18^. We show here that such a model emerges if one complements the standard model for the representations by grid and place cells by a simple self-supervised learning process for an inverse model, which learns during active locomotion to map small changes in grid-cell activity to local movement commands that caused them. This inverse model can be implemented by a weight matrix **W** (Fig. 1a). It is analogous to numerous kinds of inverse model that have previously been used for modelling biological motor control^24,25^.

Learning of **W** during exploration of the 2D space (Fig. 1c) can be implemented by a simple local plasticity rule for synaptic connections from grid cells to action-generating neurons. Due to the approximate linearity of the representation of the 2D space by grid cells and the linearity of **W**, one can use this learnt map from state changes to action commands also for selecting a first action that moves into the direction of a distant goal s*, (Fig. 1b) and the underlying theory presented in ref. ^26^. In addition, if this action selection is subject to noise, a model for goal-directed stochastic sampling of trajectories emerges. Specifically, the **W** matrix maps the difference between grid-cell codes (${{\bf{s}}}^{* }-{\widehat{{\bf{s}}}}_{t}$) for a given goal and the current location onto a superposition (*a*) of basic action commands that would cause, if executed, a movement step from the current location into the direction of the goal. If one replaces the grid-cell representation of the next location that would result from this movement step by an estimate that is produced by the forward model, one can iterate this process, yielding an imagined sequence of steps in the direction of the goal. The variance of these imagined trajectories depends on the level of noise that is superimposed on each action selection (Fig. 1d). The diversity of trajectories to the given goal that are generated in this way by the model is in the high-noise regime qualitatively similar to the one shown in fig. 4b of ref. ^18^. This predicts that the underlying level of noise in the hippocampus during the generation of imagined trajectories is fairly high. Figure 1e shows the spiking activity of place cells (based on the input that they receive from grid cells according to the architecture shown in Fig. 1a). More precisely, the underlying spike trains that generate the red-coloured trajectories in Fig. 1d are shown. They correspond to recorded spike sequences of place cells in replay studies^35^, thereby providing a link to biological data. However, place cells or spiking neurons are not required for the functioning of our model.

Importantly, the goal-directed sampling of our model has an inherent generalization mechanism, allowing the generation of imagined movement trajectories that traverse parts of the space never encountered during training, as observed in the rodent^36^. During learning of **W**, a large part of the 2D space was not accessible (Fig. 1c). Yet, the sampled trajectories can pass through it, as shown in the plots for high noise levels (Fig. 1d).

Finally, the model includes the contribution of hippocampal object cells^37^ and barrier cells^38^, whose firing encodes the distance and direction of an obstacle or wall. We model the contribution of these cells as repulsive forces from obstacles and walls during the stochastic action selection. As a result, the sampled goal-directed trajectories avoid objects and walls (Fig. 1f). Note that in this simulation, the path requires temporally moving away from the goal vector to avoid the obstacle, yet the selected paths maintain a sense of direction towards the goal. This feature becomes even more prominent when larger detours are required (Supplementary Fig. 1). This model provides a potential explanation for the puzzling finding that hippocampal replay trajectories show flexible rerouting around novel barriers, without changes (remapping) in place fields^21^. In fact, the goal-directed trajectories that the model generates look remarkably similar to the recorded ones in that study (fig. 3a of ref. ^21^).

To sum up, we have shown that stochastic sampling from a standard model for a cognitive map of 2D space supports the goal-directed imagination of trajectories from start to goal locations, capturing key qualitative patterns of rodent hippocampal replay in the same setting, including generalization to unexplored parts of space, and flexible adjustment to novel contingencies. In the following sections, we will examine whether sampling from cognitive maps can also reproduce goal-directed imagination and planning in non-spatial environments.

### Sampling from learnt cognitive maps for abstract concept spaces enables generic problem-solving through goal-directed imagination

**Fig. 2:**
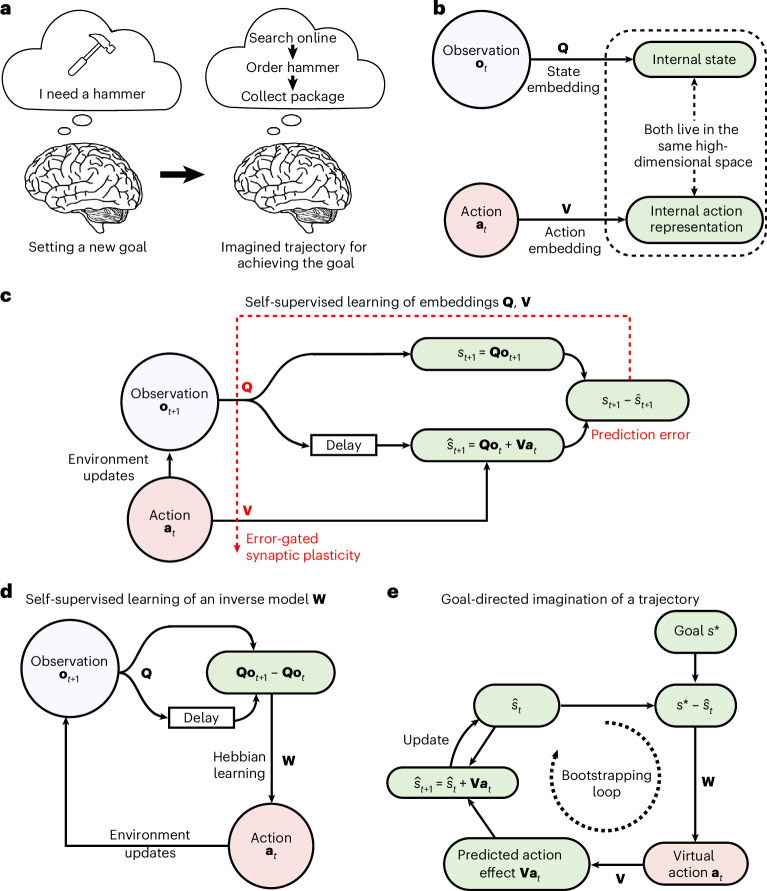
Architecture and learning process of the GCML. **a**, Illustration of a non-spatial problem that brains are able to solve through goal-directed imagination: a plan can be imagined for reaching any given goal, such as getting a hammer. **b**, The GCML uses the same cognitive-map-learning strategy as the CML model of ref. ^26^: the cognitive map arises by learning embeddings of observations and (efferent copies of) actions into a common high-dimensional state space so that the geometrical structure of the resulting cognitive map (embeddings) captures the relations between observations and actions. These embeddings **Q** and **V** can be chosen to be linear for the tasks that we consider, and can be learned by simple local rules for synaptic plasticity. **c**, Learning of the cognitive map, that is, of the embeddings **Q** and **V**, is driven by a prediction learning goal: predicting the embedding of the next observation (that is, the next state) in terms of the current observation and action according to equation (11). **d**, Simultaneously, an inverse model in the form of a matrix **W** can be learnt that maps state differences onto actions that caused them. Note that if the learnt embedding **V** (the forward model) is linear and if equation (11) is satisfied, the existence of a linear inverse model is guaranteed. **e**, To generate imagined trajectories towards a target state s*, the estimated current state ${\widehat{s}}_{t}$ is iteratively updated. However, importantly, no actions are carried out. Rather, predictions ${\widehat{{\bf{s}}}}_{t+1}$ of resulting next observations (states) replace actual feedback from the environment. The next action is virtually selected for the next predicted state (bootstrapping). The difference between a goal state s* and the estimated current state ${\widehat{{\bf{s}}}}_{t}$ drives action selection through the learned matrix **W** like for real action selection, thereby enabling goal-directed imagination.

To allow navigation in (potentially) high-dimensional conceptual spaces to solve abstract problems (Fig. 2a shows an illustration), we use a generic model for learning cognitive maps, the CML of ref. ^26^ that is based on the commonly accepted principle of predictive coding. As for cognitive maps that are created through the rodent grid-cell system, the key principle of the CML is to learn a forward model that relates current actions to the neural codes for the resulting states or observations. This is achieved in the CML model by learning embeddings **Q** and **V** of observations and actions into a common high-dimensional space (Fig. 2b). In biological terms, this high-dimensional space models the space of neural activity patterns of neurons that encode places within the cognitive map. Hence, in the brain, this coding space has a dimension that is equal to the number of neurons in that area. However, a much smaller dimension—of around 1,000—suffices for the tasks considered in this study. Similarly, it suffices to use linear maps **Q** and **V** that embed sensory inputs and efferent copies of action commands into this high-dimensional coding space. Furthermore, simple local synaptic plasticity rules suffice for learning these embeddings in a self-supervised manner during exploration (Fig. 2c). These local plasticity rules (equations (12) and (13)) approximate gradient descent for learning state predictions according to ref. ^26^. If nonlinear embeddings would be more useful, linear read-outs from fixed nonlinear circuits, as proposed by ref. ^39^, could be trained equally well. A detailed description of the CML algorithm is provided in the ‘Details of the CML algorithm’ section and Supplementary Section D.

Note that the CML is a deterministic model that interacts with the environment in an online manner, receiving a resulting observation from the environment after each action execution. To apply this model to the generation of imagined action sequences in which no observations are received, we replace observations by predictions of resulting next states (Fig. 2e). Specifically, after generating a virtual action command **a**_*t*_, the model does not receive any observation **o**_*t*+1_, and replaces it by an internally generated prediction ${\widehat{{\bf{s}}}}_{t+1}={{\bf{s}}}_{t}+{{\bf{Va}}}_{t}$. It continues from there to generate the next virtual action. Using this mechanism permits generating, for any given initial state **s**_*t*_ and any given goal state s*, an imagined trajectory **s**_*t*_, ${\widehat{{\bf{s}}}}_{t+1}$, ${\widehat{{\bf{s}}}}_{t+2}\ldots$ in the direction of the goal s*, without receiving any observation. This becomes a probabilistic generative model if one adds noise in this imaginary trajectory generation process. Specifically, we have added random values from a Gaussian distribution to the current estimate of the eligibility of each action. We call this probabilistic generative model a GCML. Note that in contrast to this generative model, the CML is a deterministic model that is not able to sample trajectories from a probability distribution. We refer to Supplementary Fig. 2 and Supplementary Section D provide a detailed algorithm and schematics of neural network implementations.

**Fig. 3:**
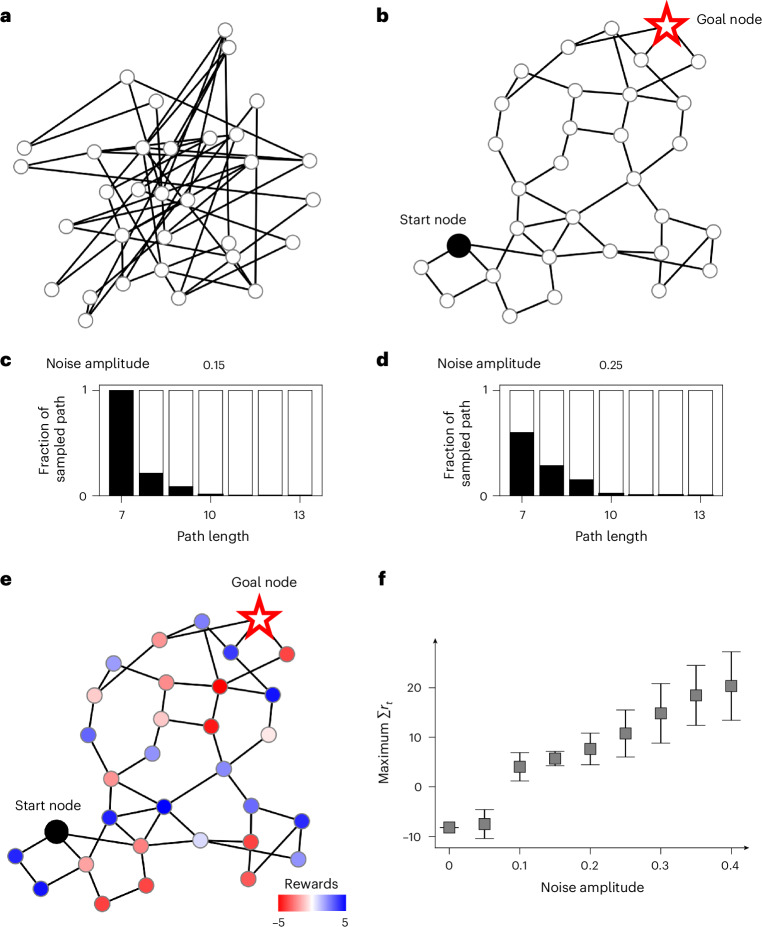
Demonstration of generic problem-solving capabilities of the GCML. **a**, A random graph with 32 nodes, which can be viewed as an abstract model for a problem-solving environment. **b**, 2D projection (via *t*-distributed stochastic neighbour embedding) of the cognitive map that the CML creates after exploring this graph with the help of its learned embeddings of observations and actions. The geometry of this graph representation supports a simple geometric heuristic strategy for selecting actions (edges) from any given node to reach any distal goal node: choose an edge that points into the direction of the goal. **c**,**d**, Two levels of noise for the length statistics of paths that are generated by the GCML for an arbitrarily fixed choice of start and goal node (indicated in **b**). For each path length indicated on the *x* axis, the black bar indicates the fraction of paths of that length from start to goal that are generated by the GCML. The lengths of paths are, in both cases, clustered around the minimum. The lower noise level in **c** provides a heuristic solution to the *k*-shortest-path problem. **e**,**f**, Illustration of a functional benefit of generating a larger diversity of solution paths (with a higher level of noise in the GCML). **e**, Possible ad hoc assignment of rewards and losses to individual nodes. If the goal is to generate a path with a maximum sum of rewards, then a higher level of noise is beneficial (**f**). For each noise level, 40 trajectories were generated by the GCML, and their maximum sum of rewards is indicated on the *y* axis. Center points indicate mean values and the error bars indicate the standard deviation over 100 times repeated experiments with different random seeds. The starting node and goal node are the same as in **b** and **e**.

As the first example for the capability of the GCML, we show that it produces not just one, but a menu of possible solutions for a problem. We use here a formalization of problem-solving that is widely adopted in AI and cognitive science: as the task to find a short path from a start to a goal node in an abstract graph—whose nodes represent problem-solving states and whose edges represent possible actions^40–43^. We consider a generic random graph with 32 nodes (Fig. 3a). Although the CML generates according to ref. ^26^, a single approximation to the shortest-path problem, the GCML provides a selection of heuristic solutions (Supplementary Section D provides the algorithmic details). They take here the form of paths from start to goal whose length is close to minimal. Such a selection of possible solutions is useful if there are other criteria besides the length, which make a particular path attractive. Mathematically, one can describe the output of the GCML as heuristic online solution to the well-known *k*-shortest-path problem^44^, where the objective is to find for some given *k*, the *k* shortest paths from a given start to a given goal. As shown in Fig. 3c, most trajectories generated by the GCML in a low-noise condition (noise of 0.15) have the shortest possible length. Higher noise levels (noise of 0.25) yield a larger diversity of solutions, some of which are slightly longer paths (Fig. 3d). However, their lengths are still clustered around the optimum. Remarkably, they still reach the goal, in spite of the higher level of noise. Even if a bad move is initially selected, it is later compensated by moving, on average, in the direction of the goal. In other words, the GCML has a self-correcting homing-in heuristic to the given goal.

Computational benefits of this capability, compared with other algorithms for solving the *k*-shortest-path problem, are exhibited in Fig. 6.

As an illustration for a functional use of having diverse heuristic solutions to a problem, Fig. 3e shows a case in which specific rewards (blue) or losses (red) are subsequently assigned to the nodes of the graph. These could encode preferences or disadvantages of using specific nodes in a solution path, such as avoiding a transfer on a trip at an airport in which a thunderstorm is reported. Figure 3f shows that higher noise levels of the GCML, corresponding intuitively to more fantasy in problem-solving, provide solution paths that collect a larger number of rewards. In this case, we let the GCML generate 40 trajectories for each noise level, and for each noise level, select the trajectory with the largest sum of rewards.

The GCML provides a neural network model for problem-solving that complements previously studied ones. With the Boltzmann machine, or via neural sampling from a spiking neural network, one could already solve problems in a less flexible manner, where all constraints and desiderata are programmed into the synaptic weights of the network^29,45,46^. By contrast, the problem-solving goal can be communicated to a GCML through synaptic input to the underlying neural network (Supplementary Fig. 2).

### Sampling from cognitive maps with compositional structure enables strong generalization of problem-solving

**Fig. 4:**
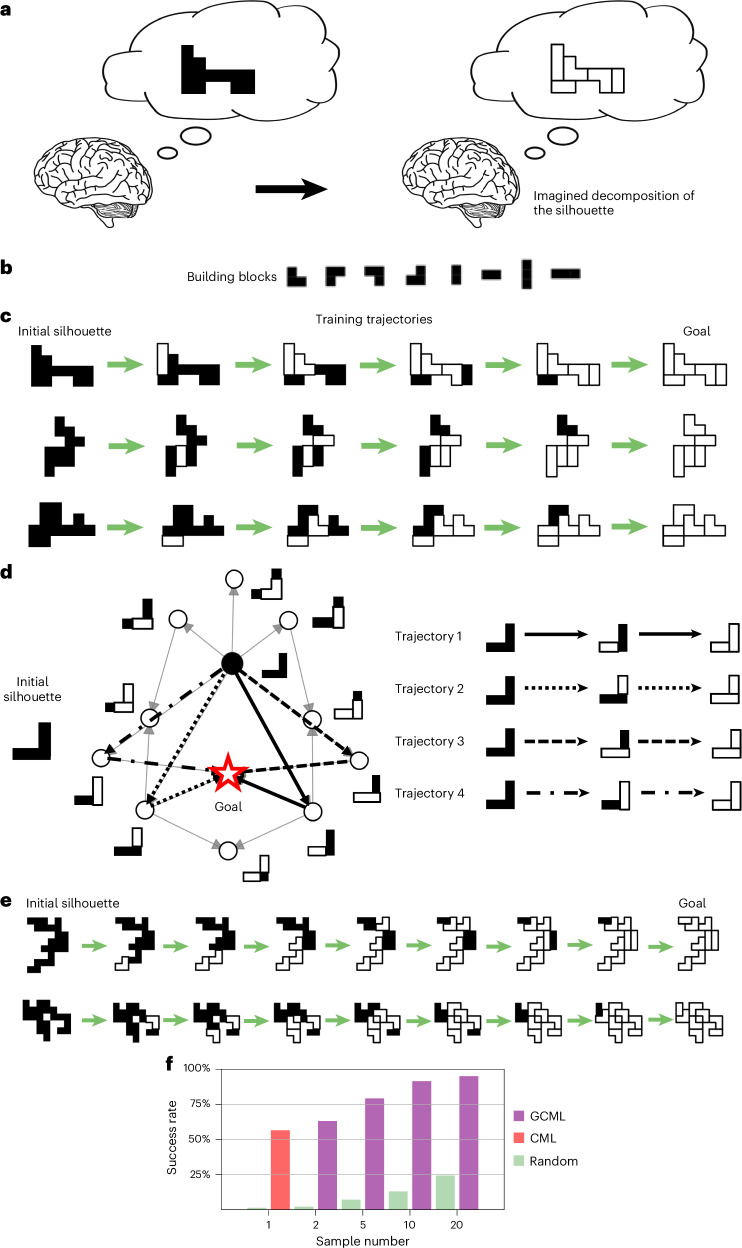
Application of the GCML to goal-directed sampling for a compositional benchmark task. **a**,**b**, Illustration of the task. Find a decomposition of the silhouette into the BBs shown in **b**, provided there exists such a decomposition. **c**, Three example trajectories that were used during learning of the GCML. The five BBs used to generate each silhouette were removed in a randomly shuffled order. **d**, 2D *t*-distributed stochastic neighbour embedding projection of the resulting cognitive map for the embedding **Q** of silhouettes with affordance factors. **e**, Two example trajectories generated by the GCML for decomposing silhouettes containing eight BBs. **f**, Test of the ability of the GCML to generate decompositions for decomposable silhouettes consisting of eight BBs, after training on the decomposition of silhouettes with five BBs. Hence, none of the test silhouettes had been encountered by the GCML before. Supplementary Fig. 4 shows additional examples.

Humans are able to imagine plans to achieve truly novel goals, such as wanting to sit together with a goat on the Schafberg mountain, once they receive a verbal (or otherwise symbolic, that is, compositional) description of such a goal. In terms of cognitive maps, such goals represent states that were never encountered before during exploration. Hence, the cognitive map structure around them could not be shaped by direct experience, and strong generalization from similar local motifs in the state space is needed. We wondered whether the GCML is able to reproduce this strong generalization capability of the human brain. We considered a benchmark task for problem-solving in a compositional domain that was recently used in a magnetoencephalography study of compositional planning processes in the human brain^22^. Participants were asked to decide whether a given silhouette (Fig. 4a, black) could be decomposed using the given set of BBs (Fig. 4b). This compositional task domain appears to be much easier than natural language processing, but it is actually NP-hard^47^, even for silhouettes that are just composed of the first four BBs shown in Fig. 4b. This implies that there is no known deterministic algorithm that can solve this decomposition task without using computational resources that grow exponentially with the size of the silhouette.

We show here that a GCML can solve through goal-directed imagination in these fairly demanding compositional tasks. A key step is that one makes the embedding **Q** of observations (here 2D silhouettes) into the cognitive map compositional. The embedding **V** of actions (here the removal of a particular BB at a particular position of the silhouette) and the inverse model **W** are learned in a self-supervised manner from successful decomposition examples (Fig. 4c). Decompositions that could be completed in five steps were used for learning. Examples are shown in Fig. 4c. The BBs that have already been removed are indicated in white with a black outline. This visual representation indicates the temporal decomposition process fairly well, but is slightly deceptive from a mathematical perspective, because the removed tiles are no longer represented in intermediate or final silhouettes. In particular, the goal state is always the same one: an empty silhouette. Hence, in this case of goal-directed sampling, the goal state is familiar, but the start state (the given silhouette) is, in general, novel.

The learnt cognitive map is for this task domain more difficult to visualize in two dimensions because of the large number of actions that can be applied to each silhouette (state). However, for a simple initial silhouette, Fig. 4d shows that the 2D projection of the cognitive map still provides a ‘sense of direction’ that favours actions that go into the direction of the target state (empty silhouette, indicated by a red star). Further examples are given in Supplementary Fig. 4. Remarkably, after learning a cognitive map from decompositions of silhouette that are composed of five BBs (Fig. 4c), the GCML is also able to decompose silhouettes consisting of eight BBs (Fig. 4e). Figure 4f shows this for a more demanding case, for various numbers of samples generated by the GCML, which outperforms a baseline algorithm (‘Random’) that removes BBs randomly and the CML model. Furthermore, the GCML shows a remarkable, near-perfect success rate, even with few samples.

**Fig. 5:**
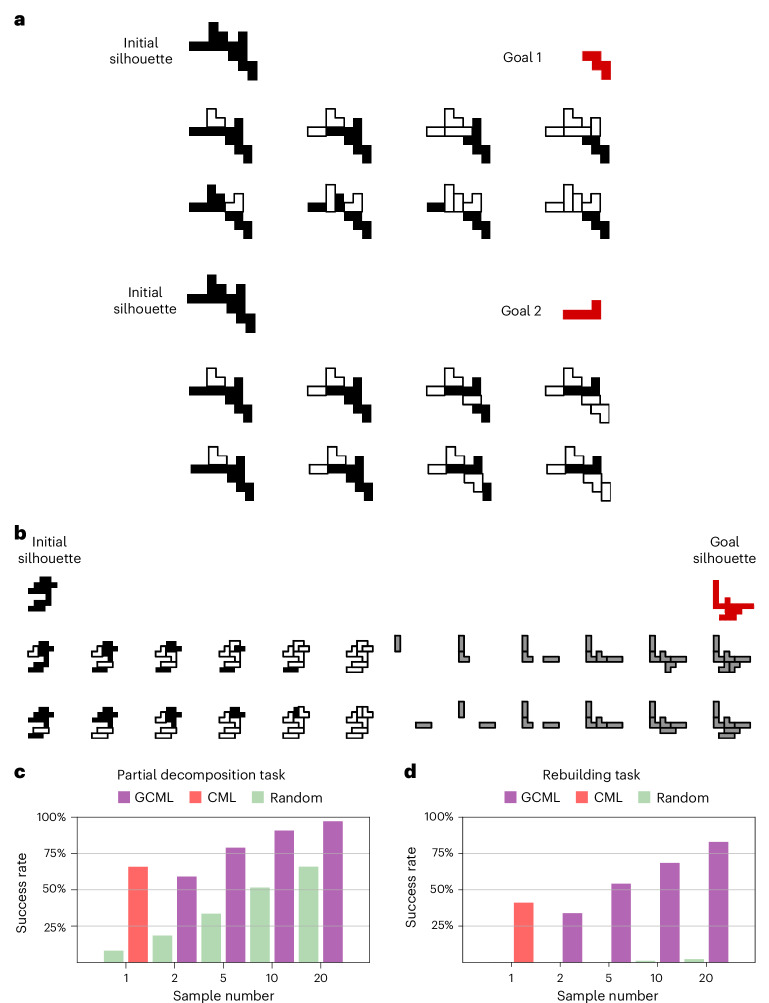
Application of the GCML for more demanding compositional computing tasks. **a**, Decomposition to a specific non-empty silhouette (containing two BBs, as shown in orange) from an initial silhouette that contains six BBs. Successful decompositions by the GCML are shown for two examples of start and goal silhouettes. **b**, Examples of trajectories generated by the GCML with the same cognitive map for a more demanding variant of the silhouette decomposition task: after decomposition, BBs have to be added to form a goal silhouette. Like the initial silhouette, it contains six BBs and, hence, did not occur during learning. The added BBs are shown in grey for two example imagined paths to the goal generated by the GCML. **c**,**d**, Evaluation of the performance of the GCML for both types of extension of the silhouette decomposition task.

Finally, Fig. 5 shows that goal-directed sampling from the same cognitive map as discussed before can also solve more general types of compositional problem. One example are problems in which the goal is not the empty silhouette, but a partial decomposition indicated as the orange goal silhouette in Fig. 5a. Figure 5c shows that the GCML can also solve this task, for silhouettes consisting of six BBs that did not occur during learning, through goal-directed sampling from the cognitive map.

Another example are problems in which BBs not only have to be removed but also have to be added, to arrive at the goal silhouette (Fig. 5b, orange). No new learning is needed for that: the action to add a BB (at a particular place of the current silhouette) is mapped to a vector that goes into the opposite direction of the one for removing this BB. Figure 5d shows that the GCML can also solve these problems fairly well, for tasks in which neither the initial silhouette nor the target silhouette occurred during learning of the cognitive map.

**Fig. 6:**
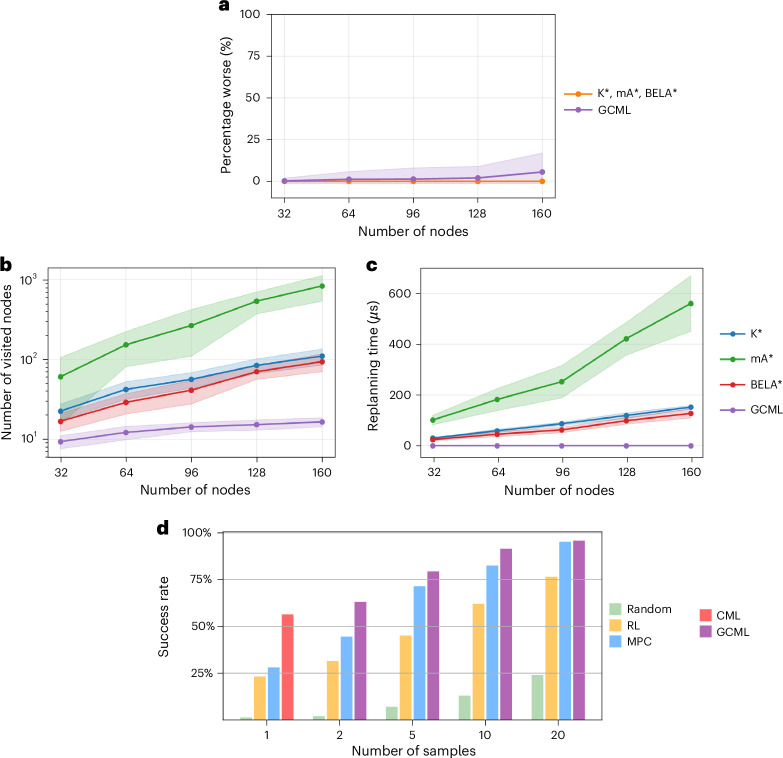
Performance comparison of the GCML with other methods for solving the *k* shortest path and tiling tasks. **a**, Performance for the *k*-shortest-path problem for random graphs with different numbers of nodes. The relative percentage worse *L*_*k*_ of the sum of lengths of the proposed *k* paths is plotted for *k* = 5. The values for K*, mA* and BELA* coincide because they produce the optimal solution. The GCML also produces close-to-optimal solutions for small graphs, with a mild degradation for large graphs. **b**, Computational effort of the four algorithms. We used the number of nodes that are visited by each algorithm to generate the first trajectory for the *k*-shortest-path problem as a measure for their computational effort. Evidently, the computational effort of the GCML is substantially lower than that of the other three algorithms. The shaded regions indicate variability across instances. **c**, Replanning latency of the four algorithms when the goal changes. The wall-clock time needed for replanning when the start or goal nodes change is shown. The GCML incurs no replanning cost, whereas the other algorithms exhibit substantially latency caused by replanning, which increases with the size of the graph. Data in **a**–**c** are presented as mean ± standard deviation across ten random graphs, with 100 random start and goal pairs per graph; the shaded error bands indicate ±standard deviation. **d**, Success rate of the GCML and four other methods for decomposing a silhouette into BBs. We consider the same task as in Fig. 4. Like in Fig. 4f, we show the success rate as a function of the number of decomposition proposals that are generated. Methods include a random policy (Random), duelling double-deep Q-network learning (D3QN; RL), CEM-based MPC (MPC), CML and GCML. The first RL bar (that is, with a sample number of 1) shows the result without any noise. The performance of the GCML is substantially better when fewer than 20 decomposition proposals are generated.

To sum up, we have shown here that endowing the cognitive map with compositional structure permits addressing challenging problem-solving tasks in compositional domains through goal-directed sampling from a cognitive map. In particular, the required solution paths had to go through never-encountered states. The compositional nature of the map, which reflects the relations between BBs and their possible combinations, permits generalizing the goal-directed imagination of the GCML not only to the decomposition of silhouettes that were never encountered during learning but also to new variants of this decomposition task: partial decomposition and combining decomposition with composition of a target silhouette. Performance comparisons with other algorithmic approaches for solving tiling tasks are given in Fig. 6. Apparently, the GCML is the only known online method for solving these tasks, in the sense that it can generate the first step towards a solution based on a sense of direction in the cognitive map, which could be viewed as some form of intuition, without taking the time to generate and examine a full solution path.

## Discussion

We have examined a simple model that integrates three prominent facets of brain intelligence: stochasticity, cognitive maps and compositional computing.

We have validated this model in three very different application scenarios. The first one (Fig. 1) reproduces biological data from the rodent brain on the imagination of possible future paths to a goal ^18,21^, including rerouting around novel barriers.

Our second application has demonstrated goal-directed sampling for problem-solving in abstract concept spaces. For this application, we used a generative extension of the CML from^26^, the GCML (Fig. 2). Figure 3 shows that the GCML provides not just a single solution to a problem but a menu of different ones.

Finally, Fig. 4 shows that our model for goal-directed sampling can also be applied in compositional task domains, where a virtually infinite number of states—most of them never visited before—can be defined by new combinations of a fixed set of components. We considered there a compositional task domain that has recently been examined through recordings from the human brain in ref. ^22^, where 2D silhouettes have to be decomposed into a given set of BBs. Figure 5 shows that the GCML can also solve demanding variations of this NP-hard compositional task, such as building a given 2D silhoutte from a given set of BBs.

Taken together, we have shown that sampling from cognitive maps provides a surprisingly powerful and versatile heuristic method to solve computationally challenging problems across both spatial and non-spatial domains, using an algorithmic form of goal-directed imagination. Of course, there are limitations to the performance of such a heuristic online method, which generates the first step of a proposed solution with low latency. More sophisticated search, reasoning and internal evaluation methods can be programmed into offline algorithms to achieve better performance and handle more difficult tasks. As indicated in Supplementary Section A, escaping local structures such as a U-shaped obstacle can require elevated noise levels, which may reduce stability. An interesting open question is to what extent this can also be achieved with brain-inspired online methods that uses simultaneously several cognitive maps on different levels of abstraction, which also include rules and memories of preceding successful solutions to similar problems (ref. ^48^ shows the first step).

In this work, we implemented a generative model by adding Gaussian noise to a deterministic (inverse) model, which corresponds to assuming a fixed uncertainty during sampling—a formulation commonly used in generative modelling (for example, ref. ^49^). Future work could explore sampling from a learned probability distribution over actions, states and goals, which might better capture the true model uncertainty. Such an approach would connect more directly to the Bayesian framework and, more specifically, to probabilistic methods for inference and planning—such as planning as inference and active inference^50–52^—in which plans are inferred from learned probabilistic transition models.

Altogether, we have introduced the GCML as a model for neural sampling that supports goal-directed sampling. Hence, it drastically enhances the application range of neural sampling in AI. In particular, it supports the drive towards more energy-efficient AI solutions for planning and problem-solving. The local learning mechanism of the GCML makes this approach suitable for self-supervised on-chip learning during exploration. Furthermore, the (virtual) action-selection mechanism of the GCML is suitable for in-memory computing with memristor crossbars, yielding a minimal delay for action selection that is independent of the size of the environment and the number of options for action selection. Hence, the GCML paves the way for creating edge devices that can use goal-directed imagination, often referred to as fantasy, to find solutions to difficult and novel tasks.

## Methods

### Details of our model for spatial goal-directed imagination

#### Generating a cognitive map based on grid cells

We used a simple cognitive map model (Fig. 1a) based on the functioning of the grid-cell system in the entorhinal cortex. Furthermore, in our applications, we derive place cells by combining grid-firing patterns.

Figure 1 shows that these experimental data can be reproduced with a standard model for the 2D spatial map that is provided by the grid-cell system of the rodent^6–8^. Furthermore, an inverse model that maps differences between grid states to movement commands that reduce this difference can be learnt by a simple local rule for synaptic plasticity. By adding noise to this action-selection mechanism, the cognitive map becomes a generative model that produces goal-directed movement plans qualitatively resembling those decoded from the rodent brain^18^, even rerouting around novel barriers, as demonstrated in the neural recordings of ref. ^21^. Furthermore, the samples generated by the model generalize to never-experienced trajectories, as observed in rodents^20,36^.

The model shown in Fig. 1a consists of grid cells, place cells and action basis neurons. All neurons were linear, with each neuron’s activation equal to the sum of its inputs. Our model is based on the core idea that grid cells can perform ideal path integration based on a velocity input^53,54^. We regard grid-cell activity itself as the state space of our model, in which both target state s* and its estimated current state ${\widehat{{\bf{s}}}}_{t}$ are expressed (Fig. 1b). We approximate post-training grid-cell activity in their model analytically, by a deterministic function ${\bf{x}}\,\mapsto \,{\bf{s}}\in {{\mathbb{R}}}^{1,000}$ that outputs the firing rates of 1,000 grid cells at any location in a 2D arena. Each grid cell is initialized independently and modelled as the sum of three positive planar cosine gratings $r({\bf{x}})={\sum }_{j=1}^{3}(1+\cos ({{\bf{k}}}_{j}\,\cdot \,{\bf{x}}+{\phi }_{j}))$, whose wavevectors **k**_*j*_ are 60° apart and share magnitude ∣**k**_*j*_∣ = 2π/*λ*. The grid scale was drawn once per cell from *λ* ≈ *U*(0.05, 8) and each phase independently from *ϕ*_*j*_ ≈ *U*(0, 2π). To eliminate directional bias, a global rotation *θ*_0_ ≈ *U*(0, 2π) was applied, giving $\arg ({{\bf{k}}}_{j})={\theta }_{0}+\mathrm{j\pi }/3$.

Under this setting, PCA applied to the grid-cell representations of the 10% largest-scale (*λ*) grid cells revealed a nearly planar 2D manifold (Fig. 1b). Because larger spatial periods generate progressively flatter interference patterns, this low-curvature embedding is well suited for goal-directed navigation.

Grid-cell firing in the brain is updated by path integration of locomotion activity. A velocity signal is provided in our model by the activity of four action basis neurons, whose sum provides a continuous velocity vector. We constrained a fixed step size *η*_*a*_ = 0.05 by normalizing the length of the resulting velocity vector always to *η*_*a*_.

To simulate continuous movement, time was discretized, with each time step representing 10 ms. The resultant movement (Δ*x*, Δ*y*) during each time step is computed by the cumulative contributions of all four action basis neurons:

1 $$\Delta x={({{\bf{a}}}_{t})}_{\mathrm{right}}-{({{\bf{a}}}_{t})}_{\mathrm{left}}\,\mathrm{and}\,\Delta y={({{\bf{a}}}_{t})}_{\mathrm{up}}-{({{\bf{a}}}_{t})}_{\mathrm{down}}.$$

This provides the forward model for the cognitive map in this section.

As in ref. ^54^, grid-cell activity can predict place-cell activity. In our model, the place fields are established by one-shot learning from grid-cell activity. The incoming weight of each place cell is assigned in a single update using a simplified rule that captures the core idea of the LTP component of behavioural time-scale synaptic plasticity^55,56^.

During 500 s of random exploration, a plateau-potential gating signal occurs every 500 ms. At each event, one postsynaptic neuron *i* is randomly selected from the neuron pool of 1,000 neurons, and its incoming weight vector **P**_*i*_ (row *i* of **P**) is overwritten with the current presynaptic grid-cell activity pattern **s**_*t*_:

2 $${{\bf{P}}}_{i}\leftarrow {{\bf{s}}}_{t}.$$

This centres the place field of neuron *i* at the location (*x*_*t*_, *y*_*t*_). Because the learning happens only at visited places, comprehensive exploration is essential; regions that are never visited cannot recruit the corresponding place fields. We used grid-cell activity, rather than place-cell firing, for generating imagined trajectories, particularly for representing the current state of an imagined trajectory. Hence, this imagination process was not affected by the lack of place cells for the unexplored region shown in Fig. 1c. Our motivation for leaving part of the 2D arena unexplored was to demonstrate that the unexplored region does not hinder grid cells from generating imagined trajectories that include segments in the unexplored region. However, to be able to plot the spiking activity of place cells during imagined trajectories (Fig. 1e), we assumed that place cells had formed in the meantime also for the initially unexplored region.

#### Using the cognitive map for generating imagined paths

An inverse model in the form of a weight matrix **W** learned in a self-supervised manner to infer actions from differences in grid-cell states which caused these differences, using the same simple Hebbian learning rule in equation (14).

We simulated here random exploration for 500 s, discretized by 10 ms within a 2D bounded environment ([0, 4] × [0, 4]), excluding the lower-left quadrant (*x* < 2, *y* < 2) shown in Fig. 1c. The agent started within the permitted region and executed a continuous-time random walk at constant speed (0.5 unit per second) with Gaussian turn noise. Invalid movements (entering forbidden zones or crossing boundaries) triggered resampling of the direction. We consistently use the matrix **W** as learned inverse model for a cognitive map throughout this paper.

We used this inverse model for (virtual) action selection during the stochastic generation of an imagined trajectories to a given goal s* as follows:

3 $${{\bf{a}}}_{t}={\eta }_{a}\,({\bf{W}}({{\bf{s}}}^{* }-{{\bf{s}}}_{t})+\epsilon ),$$

where *ϵ* is the noise that introduced stochasticity into the model, and *η*_*a*_ = 0.05 is the step size. Each dimension of *ϵ* was sampled from a Gaussian distribution ${\epsilon }_{i} \sim {N}(0,{\sigma }_{{\rm{n}}})$, where *σ*_n_ represents the noise level depicted in Fig. 1d. We assumed four basis action neurons defining an action vector ${{\bf{a}}}_{t}=[{({{\bf{a}}}_{t})}_{{\rm{up}}},{({{\bf{a}}}_{t})}_{{\rm{down}}},{({{\bf{a}}}_{t})}_{{\rm{left}}},{({{\bf{a}}}_{t})}_{{\rm{right}}}]$.

Spike generation (Fig. 1e) was modelled by interpreting the continuous activation of each place cell *i* as a firing probability *p*_*i*_(*t*) within a discrete time window of 10 ms. Spikes were then sampled from a Bernoulli distribution:

4 $${s}_{i}(t)\sim {\mathrm{Bernoulli}}\left({p}_{i}(t)\right).$$

The firing probability at each time step was computed by applying the softmax function to the normalized activation *ℓ*_*i*_(*t*) of all place cells:

5 $${p}_{i}(t)=\frac{\exp \left({\ell }_{i}(t)\right)}{{\sum }_{j=1}^{N}\exp \left({\ell }_{j}(t)\right)}.$$

#### Obstacle avoidance in imagined trajectories

For generating imagined trajectories to a given goal in the presence of obstacles (Fig. 1f), we augmented equations (3) and (1) with additional repulsive forces originating from each obstacle (*x*_o_, *y*_o_) towards the agent (*x*, *y*). The information encoded by the vector difference, **δ** = (*x*_o_ − *x*, *y*_o_ − *y*), may be represented, in the brain, by object vector cells^57^. Each wall was discretized into three point obstacles (the center and two end-points), each exerting a repulsive force **f**_*i*_. The total repulsive force **F** was the vector sum of these:

6 $${{\bf{f}}}_{i}=\frac{{{\boldsymbol{\delta }}}_{i}}{| {{\boldsymbol{\delta }}}_{i}{| }_{2}^{2}},\,{\bf{F}}=\mathop{\sum }\limits_{i}{{\bf{f}}}_{i}.$$

The denominator $| {{\boldsymbol{\delta }}}_{i}{| }_{2}^{2}$ ensures that the strength of each repulsive force diminishes with increasing obstacle distance. The total force **F**, scaled by factor *γ*, modified the movement of the agent during one simulation time step (10 ms), from equation (1) to

7 $$\Delta {x}^{{\prime} }=\Delta x+\gamma {{\bf{F}}}_{x},\,{\rm{and}}\,\Delta {y}^{{\prime} }=\Delta y+\gamma {{\bf{F}}}_{y}.$$

For all four navigation tasks in the presence of obstacles in Fig. 1f, we set *γ* = 0.2 and noise scale *σ*_n_ = 0.3.

This provides a substantially more parsimonious model for planning 2D navigation in the presence of obstacles than the model of ref. ^58^, where first, the outer product of grid and object vector cells has to be created through an offline learning process. Our model is much simpler, and predicts that no new map has to be learnt when an obstacle is moved.

### Details of the CML algorithm

The CML algorithm^26^ maps observations and actions into the same high-dimensional space. Specifically, each observation **o**_*t*_ is mapped using a learned embedding matrix ${\bf{Q}}\in {{\mathbb{R}}}^{{n}_{{\rm{s}}}\times {n}_{{\rm{o}}}}$:

8 $${{\bf{s}}}_{t}={\bf{Q}}{{\bf{o}}}_{t},$$

where *n*_s_ is the dimensionality of the state space, and *n*_o_ is the dimensionality of the observation **o**_*t*_. The target state (goal) is obtained as the embedding of a target observation:

9 $${{\bf{s}}}^{* }={\bf{Q}}{{\bf{o}}}^{* }.$$

Similarly, all possible actions **a** are embedded into the same high-dimensional space via a learned embedding matrix ${\bf{V}}\in {{\mathbb{R}}}^{{n}_{{\rm{s}}}\times {n}_{{\rm{a}}}}$, where *n*_a_ is the dimensionality of the action space. The objective of learning these embeddings is ensuring that the predicted next state ${\widehat{{\bf{s}}}}_{t+1}$ is given by

10 $${\widehat{{\bf{s}}}}_{t+1}={\bf{Q}}{{\bf{o}}}_{t}+{\bf{V}}{{\bf{a}}}_{t}.$$

Learning aims at ensuring that ${\widehat{{\bf{s}}}}_{t+1}$ becomes a good approximation of the actual next state **s**_*t*+1_ = **Qo**_*t*+1_:

11 $${\bf{Q}}{{\bf{o}}}_{t+1}\approx {\bf{Q}}{{\bf{o}}}_{t}+{\bf{V}}{{\bf{a}}}_{t}.$$

The prediction error $({{\bf{s}}}_{t+1}-{\widehat{{\bf{s}}}}_{t+1})$, which the learning rules for **Q** and **V** aim to minimize (Fig. 2c), therefore, has the following form:

12 $${{\boldsymbol{\Delta }}\bf{V}}_{t+1}={{{\eta }}}_{v}\cdot ({{\bf{s}}}_{t+1}-{\widehat{{\bf{s}}}}_{t+1}){{\bf{a}}}_{t}^{{\rm{T}}},$$

13 $${{\boldsymbol{\Delta }}{\bf{Q}}}_{t+1}={{{\eta }}}_{q}\cdot ({\widehat{{\bf{s}}}}_{t+1}-{{\bf{s}}}_{t+1}){{\bf{o}}}_{t}^{{\rm{T}}},$$

where *η*_*v*_ and *η*_*q*_ are the learning rates. These plasticity rules, known as delta rules, approximate gradient descent to minimize the prediction error. If this learning process is successful and ${\widehat{{\bf{s}}}}_{t+1}$ becomes a good approximation of the actual next state **s**_*t*+1_, one has a basis for generating a sequence of virtual actions in a goal-directed manner, without receiving observations (Fig. 2e).

In our implementation, the CML additionally learns an inverse model during exploration in the form of a matrix ${\bf{W}}\in {{\mathbb{R}}}^{{n}_{{\rm{a}}}\times {n}_{{\rm{s}}}}$. This matrix is acquired through Hebbian learning and maps state differences **s**_*t*+1_ − **s**_*t*_ onto the actions *a*_*t*_ that caused them (Fig. 2d):

14 $$\Delta {{\bf{W}}}_{t+1}={\eta }_{w}\cdot {{\bf{a}}}_{t}{({{\bf{s}}}_{t+1}-{{\bf{s}}}_{t})}^{{\rm{T}}},$$

where *η*_*w*_ is the learning rate. To every possible action **a**_*t*_, **W** assigns a utility that scores its estimated usefulness (or value) for reaching s* from the current state **s**_*t*_:

15 $${{\bf{u}}}_{t}={\bf{W}}({{\bf{s}}}^{* }-{{\bf{s}}}_{t})={\bf{W}}{{\boldsymbol{\Delta }}}_{t}.$$

Each dimension stores the utility of a specific action, forming a utility vector **u**_*t*_ of length *n*_a_. The estimation of action utility is referred to as principle 2 in CML^26^.

This mechanism relies on the hypothesis that the geometry of the learnt cognitive map provides a sense of direction when choosing the next action. Although **W** is trained only on local state differences **s**_*t*+1_ − **s**_*t*_, it is applied during planning to imaginary state differences s* − **s**_*t*_ that can be substantially larger. Linearity of the inverse model supports this generalization. These utility estimates are analogous to value estimates in RL^23,59^. In contrast to RL, however, the learned matrix **W** provides a universal value function that assigns values for reaching any possible state, and these estimates do not depend on an a priori chosen policy.

As in RL, utility estimates must be modulated by an affordance factor that vetoes actions that cannot be executed in the current state, or that increases or decreases the utility of actions depending on their difficulty in more complex environments. The utility values are masked by the learned affordance factor ${\widehat{{\bf{g}}}}_{t}\in {{\mathbb{R}}}^{{n}_{{\rm{a}}}}$, which aims to approximate the real affordance **g**_*t*_ defined by the constraints of the environment, a one-hot vector that indicates which actions are executable (1 for executable and 0 otherwise). This gating factor prevents the GCML from proposing infeasible actions. We denote the product of the utility of an action (for reaching s*) with this affordance factor as its current eligibility. Action selection at time *t* then consists of choosing the action **a**_*t*_ with the highest eligibility.

After executing this action, the CML transitions to the next state **s**_*t*+1_, represented by the embedding of the next observation **o**_*t*+1_ (**s**_*t*+1_ = **Qo**_*t*+1_). From this new state, the same action-selection procedure is iterated until the goal state is reached.

After learning **Q**, **V** and **W**, the CML can be tasked with reaching an arbitrary goal state s* = Qo* that results from a desired observation o*. Such a target o* may correspond to a spatial goal location or, more generally, to a problem to solve in an abstract state space (Fig. 2a and ref. ^26^). The learned matrix **W** is then used to select actions that are likely to be useful for reaching this goal state s*.

### Mathematical description of the GCML and its relation to biology and other theoretical models

#### Cognitive maps for non-spatial task domains

Experimental data from neuroscience and cognitive science suggest that the brain—especially the human brain—uses cognitive maps not only for spatial navigation but also for navigation in more abstract concept spaces in which spatial locations are replaced, for example, by images or by combinations of learnt ranks of an item in different linear orders^2,12–16^. We show here that the principles that enable according to the preceding section goal-directed imagination for 2D spatial cognitive maps generalize to cognitive maps for abstract concept spaces, and enable generic problem-solving (Fig. 2a shows an illustration of a non-spatial problem-solving task).

The role of the grid-cell system in supporting the generation of cognitive maps and conceptual navigation in abstract concept spaces is now well documented across a growing body of studies, including non-invasive neuroimaging experiments in humans (functional magnetic resonance imaging/magnetoencephalography^60–62^), functional magnetic resonance imaging studies in monkeys^63^ and intracranial recordings in monkeys^64^. However, the computational mechanisms that would allow grid-cell systems to scale to abstract spaces—particularly those that are not well captured by 2D Euclidean geometry and may require higher-dimensional representations—remain more debated^7^.

We used distinct maps to navigate physical space and abstract problem spaces. However, it is important to note that our approach to ‘sampling from cognitive maps’ is general and can be applied across both physical and abstract spaces, provided that the cognitive map is adapted to the task and its dimensionality. Spatial navigation and foraging occur in approximately Euclidean spaces, requiring simple 2D maps. Such low-dimensional maps can also potentially be used to address simple navigation and foraging tasks in 2D abstract spaces, such as the one studied in ref. ^60^.

However, 2D maps are insufficient for planning in non-planar random graphs and for our compositional problem-solving tasks. Hence, a system like the GCML, capable of learning in high-dimensional spaces, is useful. The use of distinct maps in our tasks is, therefore, not motivated by the difference between physical and abstract spaces, but by the dimensionality of the problems.

Crucially, by highlighting the differences between learning maps of simpler (Euclidean and 2D) versus more complex (higher-dimensional) spaces, we are not ruling out the possibility that the grid-cell system also generalizes to maps in higher dimensions required for challenging abstract tasks. For example, ref. ^7^ proposes that the grid-cell system can generalize to non-Euclidean environments with complex transition structures, using methods called ‘mixed modular coding’ or ‘map fragmentation’ (refs. ^5,65,66^ discuss other approaches).

By providing a general approach to sampling from cognitive maps, our method is agnostic as to whether the maps are learned through the grid-cell system (an idea that is increasingly empirically supported) or with the aid of other neural systems.

#### Sampling from cognitive maps for non-spatial task domains by the GCML

From a biological perspective, the mechanism used by the GCML—the stochastic generation of multiple paths and their subsequent selection based on expected reward—could be associated with the neural circuit formed by the hippocampus and the ventral striatum. Various studies suggest that this circuit supports goal-directed navigational planning and ‘vicarious trial-and-error’, with the hippocampus generating candidate future trajectories and the ventral striatum assessing the reward associated with those trajectories^67–70^.

The GCML uses the same cognitive maps as those that are learned in a self-supervised manner by the CML presented in ref. ^26^. However, the agent cannot directly access the current state during imagination from the observation. Therefore, the imagined state ${\widehat{{\bf{s}}}}_{t}$ is used as a substitute for the actual state **s**_*t*_ when computing the utility:

16 $${{\bf{u}}}_{t}={\bf{W}}\left({{\bf{s}}}^{* }-{\widehat{{\bf{s}}}}_{t}\right).$$

The computation of the imagined state will be detailed in the following paragraphs.

Another difference is that GCML cannot get the environment input on affordance, but the agent needs to learn and imagine it. The affordance in GCML is computed using the affordance gating matrix ${\bf{G}}\in {{\mathbb{R}}}^{{n}_{{\rm{a}}}\times {n}_{{\rm{s}}}}$ that receives the (imagined) state vector as its input. The matrix is updated during the learning process according to the following rule:

17 $${\boldsymbol{\Delta }}G={{{\eta }}}_{g}\cdot ({{\bf{g}}}_{t}-{\bf{G}}{{\bf{s}}}_{t}){{\bf{s}}}_{t}^{{\rm{T}}},$$

where *η*_*g*_ represents the learning rate.

The resulting vector, after applying the affordance gating, is termed the eligibility:

18 $${{\bf{e}}}_{t}={\widehat{{\bf{g}}}}_{t}\odot ({{\bf{u}}}_{t}+\epsilon ),$$

where **ϵ** is the noise is a crucial term in GCML that turns the CML into a probabilistic generative model. This injected noise enables GCML to explore actions beyond the one with maximum eligibility. Hence, in contrast to CML’s deterministic selection, GCML stochastically generates diverse trajectories.

Finally, the selected action **a**_*t*_ is the one with the highest eligibility:

19 $${{\bf{a}}}_{t}={\rm{WTA}}({{\bf{e}}}_{t}),$$

where WTA(⋅) denotes the winner-take-all operator, selecting the action with the highest eligibility from all possible actions. It can be approximated by a simple neural circuit with lateral inhibition^71^.

After selecting a virtual action **a**_*t*_ at the estimated state ${\widehat{{\bf{s}}}}_{t}$, the estimated next state, *t* + 1, is calculated using the bootstrapping process:

20 $${\widehat{{\bf{s}}}}_{t+1}={\widehat{{\bf{s}}}}_{t}+{\bf{V}}{{\bf{a}}}_{t}.$$

By iteratively applying the trajectory generation process described earlier, a virtual goal-directed trajectory is generated.

By repeatedly generating trajectories in the presence of inherent noise in equation (18), different trajectories are generated. This results in a set of possible solutions from the initial state **s**_0_ to the goal s*.

A downstream network could select a specific one from this repertoire of imagined trajectories based on a given criterion. For example, when rewards are associated with specific states, the estimated rewards ${\widehat{r}}_{t}$ of each state can be accumulated over an entire imagined trajectory, and the one with the largest sum of predicted rewards can be selected (Fig. 3f).

#### GCML suggests a model for neural sampling

On a more abstract algorithmic level, the GCML provides an intriguing paradigm for using the inherent noise of biological and physical computing systems as a computational resource^72^. An important feature is that this noise enables goal-directed sampling, for a goal that is provided in the form of a synaptic input, rather than just sampling from a fixed probability distribution that has been programmed into the weights of synaptic connections. This endows the neural sampling method with a brain-like flexibility in adjusting to new goals or contingencies, and to solve new problems that were never encountered before. From a more general theoretical perspective, our model for goal-directed sampling is related to previous models for probabilistic inference in the brain for perception and action selection through sampling^33,52,73–76^. However, in contrast to this preceding work, it provides a neural network paradigm for sampling from a marginal distribution, relative to problem specifications that are defined through the current synaptic input to the network. A theoretical basis for that had been provided in ref. ^45^. It was shown there that a stationary distribution exists for this sampling from a marginal distribution even if the synaptic connections of the network are not symmetric, which is always the case if one has synaptic connections between excitatory and inhibitory neurons. It also exists if the neurons are spiking, and exhibit a brain-like diversity of firing properties.

From a biological perspective, our approach is supported by experimental evidence, showing a dual role of (hippocampal) cognitive maps, for learning states in an associative mode and supporting sequential activity in a predictive mode^30^—and provides a possible mechanistic explanation of this finding. Novel empirical predictions can be addressed in future studies. In particular, our model proposes that together with each cognitive map, the brain also learns an inverse model that maps state differences to possible actions for reducing this state difference. Note, however, that an inverse model for a cognitive map could also be implemented in the brain in a somewhat different way, where the current and goal states are provided separately as inputs, instead of their difference. However, as long as the inverse model is approximately linear, it supports local actions with ‘foresight’, preferring actions that move into the direction of the goal.

#### Predictions of model tests through biological experiments

Future studies could align this proposal with empirical findings showing that goal and reward information shape trajectory representations and the geometry of cognitive maps in the hippocampus and cortex^77–84^. The assumption that goal-directed behaviour is driven by minimizing the discrepancy between current and goal states is shared with frameworks such as cybernetics^85–87^ and active inference^52^.

A key prediction of the GCML model for future biological experiments is that noise in the selection of possible actions during imagination is directly related to the resulting diversity of action plans, and implicitly also on the difficulty of solving a given task. This could be tested experimentally.

Furthermore, our model predicts that the speed of the generation of the next step of a multistep solution plan depends only mildly on the current distance to the goal. The reason is that the next step is generated by a feedback circuit (Fig. 2e) whose processing speed is independent of the distance to the goal. However, the generation speed of the next step is predicted to grow with the number of action alternatives that are available, since this is likely to affect the speed of the underlying WTA computation for each action selection.

Finally, our model predicts that current goals can be decoded from brain areas that are involved in action selection, as already partially shown in ref. ^83^. By contrast, RL models predict that the values of adjacent states are the most relevant for action selection, and the identity of the current goal has no direct influence.

#### GCMLs and RL

Sampling from a marginal distribution provides an alternative to RL for generating goal-directed behaviour. Models based on RL often require methods from machine learning for training, such as backpropagation and backpropagation through time, whose biological viability is debated. Furthermore, except for methods based on successor representations, RL approaches require retraining for each new goal that is to be reached. The approach of ref. ^65^ used the successor representation for RL in combination with a cognitive map. We are not aware of efforts to sample from this data structure. It is also an open problem whether this approach can be applied to tasks such as the compositional tasks that we consider, where planning needs to visit states that were not encountered during exploration.

This also holds for single-goal-conditioned contrastive RL, which was recently proposed^88,89^. However, this approaches focuses, like the CML and GCML, on learning state representations that support fast action selection. In fact, actions are selected there also through a WTA operation applied to a dot product of vectors, one of which represents the goal. The focus of single-goal-conditioned contrastive RL is on producing efficient exploration strategies, however, for a fixed goal. An open question is whether this method could also be used to enhance goal-invariant exploration in cognitive-map-based approaches.

Apart from the versatility advantage of cognitive-map-based methods, there are also some recent experimental data that suggest that dopamine signals support the learning of various types of prediction in the brain^90–92^, not only reward predictions. Hence, they might also support the learning of cognitive maps by minimizing prediction errors for sensory inputs, which provides the basis for the CML and GCML model. Altogether, the diversity of data on dopamine signals suggests that the brain could use not just one but a multitude of mechanisms and strategies for learning goal-directed behaviour, as suggested by ref. ^91^.

#### GCMLs and other theoretical models

There are various models for how the brain could learn cognitive maps, with a particular emphasis on the hippocampal–entorhinal system. Some approaches, such as Tolman–Eichenbaum machine^66^ and others^93^, emphasize a separation between learning the general structure of the environment (putatively via a grid code)—for example, the transitions between spatial locations, which generalize across different mazes—and learning the content of specific locations (putatively via place cells), which is maze specific. Other models dispense with this distinction^3,5^ but are still able to learn, offline, the transition structure of complex, non-Euclidean spaces.

The GCML, by contrast, can learn cognitive maps in high-dimensional spaces through a simple online, predictive learning rule. A unique feature of the GCML (and its predecessor, the CML) is that the learned embeddings provide a sense of direction toward goals—a capability not explicitly addressed in the aforementioned methods or related approaches.

Model predictive control (MPC) is another already-existing framework for action selection based on a learned dynamics model. Compared with the GCML, it has the advantage that it can also handle continuous state and action spaces. Apparently, however, it lacks the capability of the GCML to choose goal-directed actions with short latency, which result from the embedding of states and actions into a high-dimensional space. A nice challenge for future research will be to combine the best of both approaches in a hybrid model.

### Further details for the application of GCML for goal-directed imagination for generic problem-solving

The task setup was a random graph with 32 nodes, where the degree of each node was uniformly sampled between 2 and 5. Observations **o**_*t*_ corresponded to nodes, and actions **a**_*t*_ represented traversing edges in a specific direction. Hence, each edge is modelled by two possible actions. Both **o**_*t*_ and **a**_*t*_ were encoded as unique one-hot vectors. The training dataset consisted of 200 randomly generated trajectories, each of length 32. During the planning process, GCML samples a set of trajectories from a given starting node to a target node.

The mappings **G** transform the estimated high-dimensional state **s**_*t*_ into the corresponding affordance factors. The affordance gating vector is a binary vector indicating available actions, where ones represent feasible actions and zeros indicate restricted actions. A linear transformation is sufficient in this setting, as the vectors **s**_*t*_ are approximately orthonormal.

Exploration was regulated by adding a Gaussian noise vector **ϵ**—with each dimension sampled from ${\mathcal{N}}(0,{\alpha }_{\epsilon })$—to a normalized unit-length utility vector. The noise amplitude *α*_*ϵ*_ was set to a default value of 0.1.

Initial values for model parameters were drawn from Gaussian distributions: ${\bf{Q}} \sim {\mathcal{N}}(0,1)$ and ${\bf{V}},{\bf{W}},{\bf{G}} \sim {\mathcal{N}}(0,0.1)$. Smaller initial values for **V** improved performance. Learning rates were set as *η*_*q*_ = 0.1 and *η*_*v*_ = *η*_*w*_ = *η*_*g*_ = 0.01. GCML demonstrates robustness to variations in learning rates, similar to the original CML, with stable training performance for *η*_*g*_ in the range of [0.001, 0.05].

Each node was assigned a reward drawn from a uniform distribution ${\mathcal{U}}(-5,5)$.

To demonstrate that GCML provides a good approximation of the top-*k*-shortest-path problem (Fig. 3c,d), 40 trajectories were generated from a given starting node to a goal node under different noise levels in the GCML. The number of distinct trajectories for each possible trajectory length (that is, the number of edges in the path from the starting node to the goal node) was computed using a breadth-first search algorithm. For each trajectory length, the proportion of distinct trajectories generated by a given noise-scale GCML relative to the total number of possible distinct trajectories serves as a measure of how well GCML approximates the top-*k*-shortest-path algorithm.

### Details of goal-directed sampling in compositional task domains

This domain is arguably more transparent than natural language, but it still exhibits a core feature of compositional computing, its computational complexity. In fact, this decomposition task is NP-hard, which implies that every known deterministic algorithm requires computational resources that grow exponentially with the problem size^40,47^. Recent experimental data suggest that the human brain solves it by addressing possible BBs in a sequential manner^22^. Their recordings from the human brain showed that during planning, participants considered the possible BBs that could be removed from the silhouette in a sequential manner, starting with the most obvious candidates. Furthermore, the authors suggested that replay sequences supported a hypothesis testing process for silhouette decomposition.

Other experimental studies demonstrate, more generally, that replay from spatial and non-spatial cognitive maps provides a neural substrate for goal-directed imagination and compositional computing. But few studies have, so far, advanced mechanistic explanations of these phenomena^34,35,58,94,95^. The silhouette decomposition problem involves finding a set of BB (tiles) that completely cover a given silhouette without gaps or overlaps. This process is called the decomposition of the given silhouette. GCML generates candidate decompositions through goal-directed sampling, similar to the previously described navigation tasks. In the simplest version of the problem, the goal node o* is defined as the empty silhouette, whereas the starting node **o**_0_ is the silhouette to be decomposed. An action is defined as removing a BB from a particular position in the remaining silhouette. Details of this process are provided below.

#### BBs

We used the same set of BBs as considered in the experiments of ref. ^22^, except that we removed one of the BBs (an atomic square tile) that would make every silhouette decomposable. Hence, there were eight predefined BBs. The first four BBs were categorized as corners, each with four possible orientations and a width of 1 pixel. The remaining four BBs were bars, including two longer types (1 × 3 and 3 × 1 pixel^2^) and two shorter types (1 × 2 and 2 × 1 pixel^2^). These eight BBs were derived from the human silhouette decomposition experiment of ref. ^22^. However, we excluded the 1 × 1 BB since each presence makes each given silhouette decomposable. GCML was tasked with imagining possible decompositions of given silhouettes, each composed exactly of five BBs.

The observation of a silhouette is encoded by a binary vector that has a 1 at each pixel position in a 2D grid space that corresponds to black in the silhouette.

The simplest possible compositional embedding **Q** of silhouettes is the identity map over these binary vectors. This provides already decent performance of the GCML. However, its performance increases substantially, and its cognitive map is endowed with a better sense of direction if the embedding **Q** slightly departs from the identity map and assigns greater weight to black pixels that are surrounded in a silhouette by white pixels. This embedding increases the utility of removing BBs that cover black pixels which protrude from a silhouette. This heuristic is beneficial since the choice of these BBs is more constrained and, therefore, less error prone. It also creates a cognitive map of the compositional domain that supports the same geometric heuristic for action selection as in the previously discussed GCML applications: choose an edge from the current node that best points into the direction of the goal (Fig. 4d). Further details and the full algorithm are provided in the Methods and Supplementary Section C.

#### Generation of training and test datasets

Observation of a silhouette was encoded by a binary vector that has a 1 at each pixel position in a 2D grid space that corresponds is black in the silhouette. Each silhouette in the dataset was created by randomly selecting five BBs and placing them within a 10 × 10 image at random positions. In total, 18,000 training samples and 2,000 test samples were generated. Any silhouette appearing in the training dataset was excluded from the test dataset, regardless of its position. Each sample represented a decomposition trajectory, in which BBs were sequentially removed from an initial silhouette. The silhouette generation process followed these rules: all BBs had to remain within image boundaries without overlapping. An initial BB was placed randomly within the image with equal probability for each position. Subsequent BBs were added adjacent to the existing silhouette, with at least one pixel adjacency to increase task difficulty. Longer adjacencies increased the number of possible removal actions, further challenging the model. In the training dataset, the five BBs composing each silhouette were removed sequentially in random order to generate training trajectories.

#### Details of actions

In the silhouette decomposition problem, each action **a**_*t*_ corresponds to removing a specific BB from a specific valid position within the 10 × 10 grid, resulting in 664 possible actions. Each action is uniquely encoded as a one-hot vector. This formulation aligns with human experiments^22^, where participants manipulate BBs using a mouse and keyboard to cover the silhouette. Since moving the same BB to different positions requires distinct movements, each BB at a different position is treated as a separate action.

#### Details of observations

In the silhouette decomposition problem, the observation ${{\bf{o}}}_{t}\in {{\mathbb{R}}}^{10\times 10}$ directly represents the silhouette image, rather than being encoded as a one-hot vector as in previous experiments.

#### High-dimensional embedding details

The simplest possible compositional embedding **Q** of silhouettes is the identity map over these binary vectors. This provides the already decent performance of the GCML. However, its performance increases substantially, and its cognitive map is endowed with a better sense of direction if the embedding **Q** slightly departs from the identity map and assigns greater weight to black pixels that are surrounded in a silhouette by white pixels. This embedding increases the utility of removing BBs that cover black pixels that protrude from a silhouette. This heuristic is beneficial since the choice of these BBs is more constrained and, therefore, less error prone. It also creates a cognitive map of the compositional domain that supports the same geometric heuristic for action selection as in the previously discussed GCML applications: choose an edge from the current node that best points into the direction of the goal (Fig. 4d). The state representation **s**_*t*_ = **Qo**_*t*_ lies in a 100-dimensional space that matches the dimensionality of the observation. In our implementation, however, this effect is achieved entirely through the context-dependent affordance term (see the paragraph describing ${{\mathsf{g}}}_{2}$), which already assigns higher influence to protruding pixels through its convolution-based computation. As a result, no explicit modification of **Q** is required, and we maintain **Q** = **I**. Further algorithmic details are provided in Supplementary Section C.

#### Details of learning the cognitive map

The action embedding matrix **V** is learned according to equation (12). The learning rate of **V** was set to *η*_*v*_ = 0.01, with values between 0.0001 and 0.1 also demonstrating stable performance. In this silhouette decomposition task, each action corresponds to removing a specific BB at a specific valid location, and the corresponding action embedding **V** is trained to predict the resulting silhouette after the removal of that block (equation (11)).

The inverse model **W** is learned in a self-supervised manner from successful decomposition trajectories using equation (14) with a learning rate *η*_*w*_ = 1, and the weights in **W** saturate at 1:

21 $${{\bf{W}}}_{t+1}=\min \left({{\bf{W}}}_{t}+{{\bf{a}}}_{t}{\left({{\bf{s}}}_{t}-{{\bf{s}}}_{t+1}\right)}^{{\rm{T}}},1\right).$$

As a result, **W** becomes a binary matrix. Both matrices **V** and **W** were initialized using Gaussian distributions ${\mathcal{N}}(0,0.001)$. The method remained effective across a broad range of initial standard deviations, from 0.0001 to 1.

One can easily see that **W** outputs for a given state difference for each possible action (BB) the number of pixels in the overlap between the state difference and the BB, provided that the BB is contained in the state difference (otherwise, it outputs 0).

#### Affordance details

The affordance gating mechanism in the silhouette decomposition problem comprises two components. The first component, ${{\mathsf{g}}}_{1}$, ensures that the agent selects only BBs present in the silhouette. We assumed GCML could directly access ${{\mathsf{g}}}_{1}$ from the environment during all the imagined steps. This assumption aligns with biological experiments^22^, where subjects directly observe the screen as they solve the silhouette decomposition problem. Thus, affordance information is assumed available at all steps, requiring no inference from ${\widehat{{\bf{s}}}}_{t}$.

The second component, ${{\mathsf{g}}}_{2}$, represents context-dependent affordance determined by the estimated state ${\widehat{{\bf{s}}}}_{t}$. Each pixel’s affordance value is set as the number of non-empty adjacent pixels (top, bottom, left and right). This is modelled as a convolution operation with the kernel:

22 $${\bf{k}}=\left[\begin{array}{ccc}0 & 1 & 0\\ 1 & 0 & 1\\ 0 & 1 & 0\end{array}\right]$$

using a 3 × 3 kernel size, padding of 1 with value 1, and stride of 1. The context-dependent affordance function ${{\mathsf{g}}}_{2}$ transforms the delta vector ${\boldsymbol{\Delta }}={{\bf{s}}}^{* }-{\widehat{{\bf{s}}}}_{t}$ into

23 $${{\mathsf{g}}}_{2}({\boldsymbol{\Delta }})=({\bf{k}}* ({\bf{1}}+{\boldsymbol{\Delta }}))\odot (-{\boldsymbol{\Delta }}),$$

where * denotes the convolution, and ⊙ represents element-wise multiplication. The delta vector **Δ** contains unremoved pixels with a value of −1 and all other pixels with a value of 0. The expression (**1** + **Δ**) flips these values, converting −1 to 0 and 0 to 1; therefore, the subsequent convolution counts the number of adjacent pixels with a value of 0. Finally, since only the affordance values at unsolved pixels are relevant, an element-wise multiplication between the minus delta vector (−**Δ**) and the affordance value **k**⋅(**1** + **Δ**) is performed as the second component of the affordance check.

#### Trying to decompose a silhouette

This task was formulated as a navigation problem, where the embedding of the empty silhouette, o* = **0**, represents the target node in the high-dimensional state space (cognitive map), whereas the embedding of the initial silhouette, **o**_0_, served as the starting node. In the presence of noise, GCML selected the action with the highest utility value from the set of currently feasible actions, determined by the environment’s affordance factors ${{\mathsf{g}}}_{1}(\cdot )$ and ${{\mathsf{g}}}_{2}(\cdot )$. This selection process followed the directional intuition of the cognitive map:

24 $${{\bf{a}}}_{t}={\mathrm{WTA}}({{\mathsf{g}}}_{1}({\bf{W}}{{\mathsf{g}}}_{2}({\boldsymbol{\Delta }}))+\boldsymbol{\epsilon} ),$$

where the noise term **ϵ** followed a Gaussian distribution ${\mathcal{N}}(0,0.1)$.

After selecting an action **a**_*t*_, the next state was estimated as ${\widehat{{\bf{s}}}}_{t+1}={\widehat{{\bf{s}}}}_{t}+{\bf{V}}{{\bf{a}}}_{t}$, and GCML proceeded to the next iteration to attempt the removal of another BB. The algorithm terminated when no feasible actions remained. If all pixels were removed, GCML successfully identified a valid decomposition; otherwise, the attempt was deemed unsuccessful.

The four paths from start to goal that are plotted in Fig. 4d with solid, dotted, dashed and dash–dotted line segments correspond to four trajectories that solve the problem. Their intermediate states are indicated in Fig. 4c (right). Note that all of these correct trajectories result from actions that move into the direction of the goal in this cognitive map. The top part of the left plot in the panel depicts three silhouettes that result from removing a ‘wrong’ BB from the starting silhouette that does not lead to a full decomposition of the initial silhouette. Note that they lie relative to the starting point on the opposite direction of the goal. Another dead-end configuration that arises if one does not move towards the goal node in the second removal step of the paths indicated by solid and dotted lines is shown at the bottom. Additional examples of cognitive maps and generated trajectories to decompose silhouettes are shown in Supplementary Fig. 4.

#### Details of performance evaluation

The performance of GCML was evaluated based on the proportion of test cases in which it successfully solved the given silhouette in the test dataset.

As baseline comparisons, we consider a random strategy that removes as many BBs as possible from the silhouette in a random manner, as well as the CML algorithm^26^.

#### Decomposition to a given non-empty goal silhouette

In the non-empty goal silhouette decomposition problem, GCML removes BBs from a given silhouette **o**_0_ to transform it into various non-empty target silhouettes o* ≠ **0**. When generating the test dataset, the initial silhouettes contained six BBs, generated by following the same method described earlier. Any initial silhouette that appeared in the training dataset was removed, regardless of its position. The goal silhouettes contained two BBs, generated by randomly removing four BBs from the six BBs used to generate the initial silhouettes. GCML could plan trajectories for non-empty goal silhouettes using equation (24) without requiring retraining. The performance was evaluated using 1,000 test silhouette pairs.

#### Combining sihouette decomposition and composition

To address the silhouette rebuilding problem, GCML began with a given silhouette and iteratively modified it by adding or removing BBs to reconstruct the goal silhouette. This task was solved using the same cognitive map learned from the silhouette decomposition problem introduced earlier, leveraging mathematical symmetry.

GCML followed a two-stage approach. In the first stage, it removed BBs as long as possible from the given silhouette using a straightforward method. Here GCML adopted for that a sub-goal **s**^†^ = **0**, and the delta vector was computed as **Δ** = **s**^†^ − **s**_*t*_. Then, it switched to the second stage, where it aimed at constructing the given target silhouette by sequentially adding BBs. More precisely, the goal was the final silhouette s* = Qo*, and the delta vector was given by **Δ** = s* − **s**_*t*_. Since adding BBs was the inverse operation of removing them, GCML selected actions based on

25 $${{\bf{a}}}_{t}^{{\prime} }=\mathrm{WTA}\left({{\mathsf{g}}}_{1}^{{\prime} }\left({\bf{W}}{{\rm{g}}}_{2}^{{\prime} }{\left({\boldsymbol{\Delta }}\right)}^{{\rm{T}}}\right)+\boldsymbol{\epsilon} \right),$$

where the affordance factor ${{\mathsf{g}}}_{1}^{{\prime} }$ ensured that GCML only added BBs contained within the goal s*. The function ${{\mathsf{g}}}_{2}^{{\prime} }({\boldsymbol{\Delta }})=({\bf{k}}* ({\bf{1}}-{\boldsymbol{\Delta }}))\odot {\boldsymbol{\Delta }}$ was designed following the same principle as in equation (23). The negative transformation of the sign of **Δ** in equation (23) facilitated the addition of BBs as the inverse of their removal. Here ${{\bf{a}}}_{t}^{{\prime} }$ represented the action of adding a BB corresponding to **a**_*t*_, which removed a BB.

For the testing dataset, the initial and goal silhouettes were generated using the five BBs following the previously introduced generation process. To test the robustness of our model, a randomly selected noise pixel was added to the same empty location in both start and goal silhouettes. Any initial or goal silhouette that appears in the training dataset was removed, regardless of its position. A total of 1,000 initial and goal silhouette pairs were generated to test the performance.

### Comparison of performance of the GCML with competing methods for solving the *k* shortest path and tiling tasks

We compared the performance of the GCML on the *k*-shortest-path problem with that of three other algorithms that have been proposed for this task: K*, mA* and bidirectional edge labelling A* algorithm (BELA*) algorithms. The results are shown in Fig. 6a–c. In Fig. 6d, we compare the performance of the GCML for tiling tasks with that of an RL algorithm and the MPC algorithm. We found that the performance of the GCML is fairly competitive, in spite of the fact that it is an online algorithm: it immediately generates the first heuristic step towards a solution, without generating a complete solution path, or even generating and comparing many possible solutions paths in an offline manner, as the competing methods do (we are not aware of competing online methods for solving this task, except for the random method examined in Fig. 6d). Our comparisons show that the GCML requires altogether much less computational effort and latency. We also expect that it is superior when fast adaptation to a variation of the task is desired.

Detailed description of the competing algorithms and details of our comparisons are given below. We also discuss the differences in the amount of working memory that is required by different approaches.

#### *k*-shortest-path problem

As shown in the main text, GCML is an approximate *k*-shortest-path algorithm. We compare it with three *k*-shortest-path algorithm baselines: the classical K*^96^, mA*^97^ and more advanced BELA*^98^. No heuristic form of the algorithms is used, since nodes in our random graphs environment are equivalent.

##### Details of K* algorithm

K* is an on-the-fly algorithm for enumerating the *k* shortest paths in a non-decreasing cost order^96^. It first runs an A*-style search to obtain the shortest path and to incrementally reveal sidetrack edges (that is, detours) relative to the current shortest path tree. These detours are organized into a path graph on which a Dijkstra-like procedure is executed to repeatedly extract the next best detour combination, reconstruct the corresponding full path and output it. The two searches are interleaved: when the auxiliary search exhausts currently known detours, K* resumes A* to expand more of the original graph and updates the auxiliary structure, continuing until *k* solutions are produced.

##### Details of mA* algorithm

mA*^97^ extends A*^99^ from returning a single shortest path to enumerating the *k* shortest paths in non-decreasing path length. It maintains a priority queue of candidate nodes initialized with the start node and repeatedly selects the most promising node according to an evaluation that combines the accumulated path length with a heuristic estimate of the remaining distance to the goal (that is, the remaining distance is fixed at zero when no heuristic information is provided). The selected node is expanded to generate successors (that is, child nodes). The algorithm discards any successor that has already been generated *k* times; otherwise, the successor is inserted into the priority queue with its updated cost. Unlike standard A*, it does not terminate after the first goal-reaching path; each time a complete start-to-goal path is found, it is output as one solution and the search continues until *k* solutions are produced.

##### Details of BELA* algorithm

Compared with mA*, BELA*^98^ does not enumerate the *k* shortest path by repeatedly pushing many alternative path-specific copies of the same state into the priority queue. Instead, BELA* keeps the forward search closer to a single A*-like exploration that builds an explored graph with sufficient predecessor information, and it delegates the top-*k* enumeration to a separate layer: it stores each newly certified deviation from the shortest-path tree as a centroid (that is, a compact representation of a family of solutions) and later reconstructs multiple complete paths at once by combining compatible prefixes and suffixes from the explored structure. Consequently, BELA* emphasizes the implicit representation of many solutions and outputs them only when their ordering among the next shortest solutions is guaranteed.

##### Measure of performance

To quantify the performance *L*_*k*_ of a *k*-shortest-path algorithm, we compute the relative error in the total length of the returned *k* paths. Let *p* denote the summed path length of the different *k* shortest paths produced by the algorithm, and let *p*_*k*_ denote the ground-truth summed path length. We define *L*_*k*_ as

26 $${L}_{k}=\frac{p-{p}_{k}}{{p}_{k}}.$$

Lower *L*_*k*_ is better.

We also report the latency to obtain the first shortest path to the goal, measured as the number of visited nodes required to generate the first trajectory. For the baselines, this metric counts how many times a node is removed from the candidate queue and expanded by generating successors and inserting them into the queue. It is a standard measure of search effort and an implementation-robust proxy for computational cost. Baseline methods typically visit far more nodes than the length of the returned trajectory because they must explore the graph. For GCML, each decision selects an affordance-feasible action and transitions to the next state, so the visited-node count equals the sampled trajectory length.

##### Comparison of performance

Here we set *k* = 5 for all experiments. We evaluate all methods on ten graphs for each node size (*N* ∈ {32, 64, 96, 128, 160}) and 100 random start and goal pairs per graph. The graph structure and the start and goal pairs remain unchanged for all algorithms. For GCML, to ensure a well-trained cognitive map at larger *N*, we fix the number of trajectories to 1,000 and the state dimensionality to 5,000; all other settings are provided in this section.

Results are shown in Fig. 6a,b. As shown in Fig. 6a, the three baselines are exact and, therefore, achieve *L*_*k*_ = 0. GCML degrades slightly as *N* increases. However, as shown in Fig. 6b, GCML visits far fewer nodes to generate the first potential trajectory of the solution than the baselines, benefiting from the directional guidance provided by the cognitive map, which allows it to avoid extensive exploration of the graph as baseline algorithms.

##### Comparison of replanning latency

We also measured replanning latency when the start or goal node changes, keeping the graph fixed. Replanning latency is defined as the wall-clock time an algorithm needs to explore the graph after the start or goal query is changed whereas the underlying graph remains fixed. All experiments related to wall-clock time in this section were run on a workstation equipped with dual Intel Xeon E5-2690 v4 CPUs (2.60 GHz), providing 28 physical CPU cores and 56 hardware threads in total, and 6 NVIDIA Quadro P6000 GPUs connected via PCIe, with 24 GB of on-board memory per GPU. As shown in Fig. 6c, the replanning time of the baseline algorithms increases with the number of nodes. By contrast, because GCML relies on the learned cognitive map, it incurs negligible additional replanning cost.

##### Comparison of working memory consumption

We define the working memory of the algorithms as the transient memory required during online trajectory generation. The three search-based baselines impose a substantially heavier online memory burden than GCML, although for different reasons. K* must preserve structures for sidetrack management and path enumeration. mA* must maintain a dynamically growing search frontier, a record of explored states and multiple predecessor references to support the recovery of alternative shortest paths. BELA* also requires a record of labelled explored state and auxiliary structures called centroids for combining prefixes and suffixes of candidate paths. As the number of requested solutions increases, these intermediate representations of the baseline algorithms can accumulate.

By contrast, GCML performs online generation by following a learned cognitive map prior and sampling directly towards the goal; therefore, its transient memory is dominated by the current local decision context and the partial trajectory being produced, rather than by a global search over the graph. Consequently, GCML’s online working memory is typically much closer to a local, near-constant-size inference buffer, whereas the baselines require graph-scale, search-dependent memory that grows with problem difficulty and the number of requested paths. In conclusion, GCML may incur a fixed static memory cost for storing model parameters and the learned cognitive map, whereas the working memory of the search baselines grows dynamically during online computation.

#### Tiling problem

We implemented two mature and widely used baselines on the same tiling environment setting used by our method (that is, five BBs in training silhouettes and eight BBs in the test silhouettes): an RL baseline and an MPC baseline.

##### Details of RL algorithm

For the RL algorithm, the duelling double-deep Q-network learning algorithm is adopted. The Q-function uses a duelling architecture^100^ with double-deep Q-network target selection^101^. The definition of observation and action remains unchanged to GCML. When selecting actions, the action feasibility is enforced by an affordance mask as in GCML (invalid actions are masked before selection by affordance). We tuned the learning rate over {10^−3^, 10^−4^, 10^−5^, 10^−6^} and selected the value with the best validation performance. We also varied the depth of the encoder’s linear layers, the value and advantage heads, adopting the best-performing architecture.

The network model consists of a convolutional encoder, and two duelling heads for value and advantage estimation. The encoder has (1) a convolutional stack with channels 1 → 32 → 64 → 64, using 3 × 3 kernels with stride 1 and padding 1, followed by rectified linear unit activations, and (2) a feed-forward stack of 6,400 → 1,024 → 1,024 → 512 with LayerNorm and Gaussian error linear unit activations, producing a 512-dimensional state representation *s*. On top of this representation, the value head is an multilayer perceptron with layer sizes of 512 → 512 → 256 → 128 → 1 that outputs *V*(*s*), and the advantage head is a multilayer perceptron $512\to 512\to 256\to 128\to | {\mathcal{A}}|$ that outputs *A*(*s*, *a*); both heads use LayerNorm and Gaussian error linear unit activations. ${\mathcal{A}}$ denotes the set of all possible actions.

We train the network with a duelling critic and experience replay. The Q-network is optimized with Adam (learning rate of 10^−5^) using mini-batches of 512 transitions sampled from a replay buffer of capacity 2 × 10^5^. We use a discount factor of *γ* = 0.99. Training is run for 10,000 episodes. Exploration follows an *ϵ*-greedy policy with *ϵ* linearly annealed from 0.30 to 0.02 over 1,200 episodes. During evaluation, to generate different trajectories, we keep a stochasticity with *ϵ*_eval_ = 0.3 (optimized among {0.05, 0.1, 0.15…0.4}). With probability *ϵ*_eval_, it performs policy-guided stochastic exploration: affordance-masked action scores from the trained Q-network are converted into a probability distribution via softmax, and a valid action is sampled from this distribution. The per-step reward is designed to reward the valid decisions: ${r}_{t}=2\cdot {{\mathbb{I}}}_{{\rm{done}}}$, where ${{\mathbb{I}}}_{{\rm{done}}}$ indicates task completion.

##### Details of MPC algorithm

To choose an action, MPC^102^ plans an optimal action sequence under a learned policy model, executes only the first action and replans at the next step by discarding the remaining actions. In our implementation, MPC uses the cross-entropy method (CEM)^103^ as its policy optimization (planning) procedure. CEM maintains a sampling distribution over action sequences and updates it iteratively over time. At each iteration, it draws candidate sequences, rolls them out through the learned dynamics to approximate the induced state trajectories and returns, and scores each candidate accordingly. A fixed number of the best-scoring sequences are then treated as elites, and their statistics are used to refit the distribution, yielding an updated action distribution for the current decision step.

Further details of the MPC algorithm are given below. The observation and action definition is same as GCML. At real step *t*, MPC optimizes an action sequence

$${{\bf{a}}}_{t:t+H-1}^{(i)}=\left({a}_{t}^{(i)}\ldots {a}_{t+H-1}^{(i)}\right),$$

for *i* = 1…*N* sampled rollouts, where *H* is the planning horizon and *N* = 96 is the sample count. We set the horizon *H* to 5 to stay consistent with training on five-BB silhouettes, and then test performance on eight-BB silhouettes.

Sampling is performed from per-time-step categorical distributions:

27 $${a}_{t+\tau }^{(i)} \sim {\pi }_{\tau }(\cdot ),\,\tau =0\ldots H-1,$$

with affordance like GCML. Here *π*_*τ*_(⋅) is categorical policy at planning step *τ*. After simulating a candidate sequence, we compute the CEM score defined as

28 $${J}^{(i)}=-\,{r}^{(i)}+\beta {\chi }^{(i)},$$

where ${r}^{(i)}=\parallel {{\bf{s}}}_{t+H}^{(i)}-{{\bf{o}}}^{* }{\parallel }_{1}$ is the terminal mismatch, ${\chi }^{(i)}={\mathbb{I}}[{r}^{(i)}=0]$ is the success flag, ${{\bf{s}}}_{t+H}^{(i)}$ is the remaining silhouette at step *t* + *H*, o* is goal silhouette and *β* = 100 is terminal success bonus. Higher *J*^(*i*)^ is better.

For each planning step *τ*, the elite empirical distribution is

29 $${\widehat{\pi }}_{\tau }(a)=\frac{{c}_{\tau }(a)+\delta }{{\sum }_{{a}^{{\prime} }}\left({c}_{\tau }({a}^{{\prime} })+\delta \right)},$$

where *c*_*τ*_(*a*) is the elite count of action *a* at step *τ*, and *δ* = 10^−6^ is the minimum action probability. Then, distribution smoothing is

30 $${\pi }_{\tau }\leftarrow (1-\alpha ){\pi }_{\tau }+\alpha {\widehat{\pi }}_{\tau },$$

followed by clamping *π*_*τ*_(*a*) ≥ *δ* and renormalization. Here *α* = 0.3 is the smoothing factor.

CEM optimization runs for *I* = 4 iterations, then only the first action of the best sequence is executed:

31 $${a}_{t}^{\star }={{\bf{a}}}_{t:t+H-1}^{\star }[0].$$

One BB is removed following ${a}_{t}^{\star }$, horizon shifts by one step, and planning repeats until success or no more BBs could be removed.

##### Comparison of performance

The success rates (defined in the same way as for GCML) for the random baseline, RL, MPC, CML and GCML are shown in Fig. 6d. All algorithms are evaluated on the same 2,000 test samples as described here.

For the RL baseline, the network architecture is much more complex than those of the other methods. It also requires backpropagation to train, which makes deployment on neuromorphic hardware challenging. In addition, the reward is difficult to design, and the sparsity of the reward signal makes training unstable.

For the MPC baseline, there is no policy training, and it achieves higher success rate than the RL baseline. However, compared with RL and GCML, MPC incurs higher planning cost because it performs an iterative sampling-and-refinement procedure at every decision step, rather than amortizing computation across episodes. In our implementation, MPC uses CEM to optimize a horizon *H* action sequence by repeatedly rolling out *N* candidate sequences for *I* iterations, which results in a per-step workload proportional to *O*(*I*⋅*N*⋅*H*). This issue is exacerbated in compositional tiling tasks in which early placements can create irreversible geometric constraints; therefore, short-horizon optimization may fail to anticipate long-term feasibility. Consequently, when the sampling budget is reduced (smaller *N* or fewer iterations *I*), MPC’s distribution updates become noisy and can collapse to suboptimal modes, leading to a pronounced drop in success rate. By contrast, GCML leverages a cognitive map that provides a global embedding of the observations and the learnt utility, which guides sampling towards regions that are more likely to yield feasible completions. Therefore, under comparable or even smaller online budgets, GCML can maintain higher success and better sample efficiency than MPC in the current setting. Overall, we emphasize that MPC remains a strong general-purpose control baseline, but its per-step compute and horizon-limited look ahead makes it less attractive for the highly combinatorial environments like the tiling problem. Same as the RL baseline, it also needs to define a rollout score equation (28) to measure the quality of a candidate action sequence.

Our GCML achieves the best overall success in this task across different sample numbers and requires algorithmically simpler local updates compared with RL and MPC.

### Reporting summary

Further information on research design is available in the Nature Portfolio Reporting Summary linked to this article.

## Supplementary information

Supplementary Information Supplementary Sections A–E, Figs. 1–4, Algorithms 1 and 2 and Discussion.

Reporting Summary

## Data Availability

All data used in the experiments were generated synthetically and are publicly available via GitHub at https://github.com/LH-cbicr/GCML and via Zenodo at 10.5281/zenodo.19370442 (ref. ^104^).

## Code availability

The code used for training and evaluating the GCML is publicly available via GitHub at https://github.com/LH-cbicr/GCML and via Zenodo at 10.5281/zenodo.19370442 (ref. ^104^).
