# Supplementary material for: Neural sampling from cognitive maps enables goal-directed imagination and planning
Source: Nat Mach Intell. 2026 Jul 21;8(7):1045–65. doi: 10.1038/s42256-026-01254-4 (PMC13395624; doi:10.1038/s42256-026-01254-4)
Supplement: Supplementary file 1 — Supplementary Sections A–E, Figs. 1–4, Algorithms 1 and 2 and Discussion. [file 42256_2026_1254_MOESM1_ESM.pdf]

nature machine intelligence

Supplementary information https://doi.org/10.1038/s42256-026-01254-4

Neural sampling from cognitive maps enables goal-directed imagination and planning

In the format provided by the authors and unedited

Table-of-Contents

Section	Item
**A.** Obstacle avoidance of goal-directed sampling from a cognitive map based on grid cells (complementing Section 2.1 and Fig. 1)	**Fig. S1** A sufficiently large noise level enables planning to escape from U-shaped obstacles
**B.** Possible implementation of the generation of cognitive maps by CMLs and GCMLs	**Fig. S2** Generation of cognitive maps for the CML and GCML through self-supervised learning
**C.** Scaling properties of the CML and GCML	**Fig. S3** Scaling of the GCML performance on random graphs
**D.** Details of the GCML planning algorithm for sections 2.2 and 2.3	**Algorithm 1** Generating Trajectories in the Abstract Graph Task<br>**Algorithm 2** Generating a Set of Possible Decompositions in the Silhouette Task
**E.** Cognitive maps for two further instances of the tiling-decomposition task considered in Section 2.3	**Fig. S4** Two further examples for the cognitive map that the GCML generates for the tiling problem

A Obstacle avoidance of goal-directed sampling from a cognitive map based on grid cells (complementing Section 2.1 and Fig. 1)

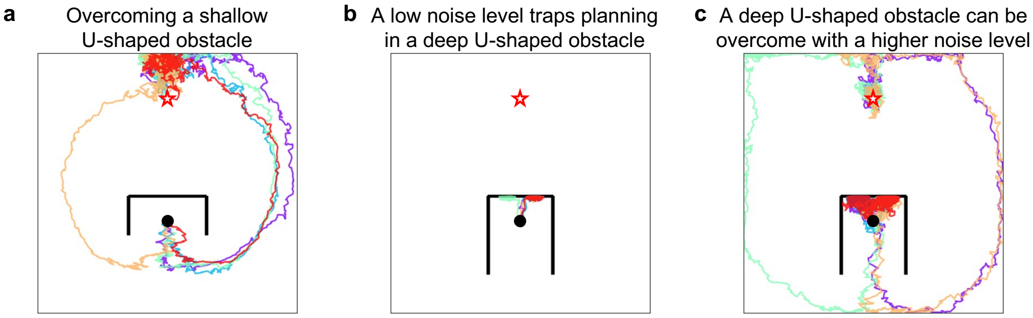

Fig. S1: **A sufficiently large noise level enables planning to escape from U-shaped obstacles.** (a) With the same noise level 0.3 as in Fig. 1f the goal-directed sampling can overcome shallow U-shaped obstacles. (b) This does not hold for a deeper U-shaped obstacle. (c) But with noise level 0.5 the same goal-directed sampling can overcome also the deeper U-shaped obstacles, and still reach the goal.

In this section, we evaluate the ability of goal directed sampling from a grid-cell based cognitive map to handle navigation tasks that require detours. As example we consider escape from U-shaped obstacles.

As described in Section 4.1, the agent's movement results from the combined effect of three components: (1) attraction toward the goal, (2) stochasticity introduced through the noise term ϵ in action selection, and (3) a repulsive force exerted by obstacles. The sum of these components determines the direction of each movement step.

Detour-based navigation, where the agent must temporarily move away from the goal, is enabled by the latter two components: noise and repulsion. Using the same parameters as in Fig. 1f, we generated the trajectories shown in Fig. S1a. When the U-shaped obstacle is shallow, the repulsive force alone is sufficient to drive the agent around the obstacle; all paths consistently move downward from the starting point.

However, this simple mechanism fails when the obstacle becomes deeper (Fig. S1b). In this case, the extended lower walls exert upward repulsive forces, which—under the simple repulsion rule used in Section 4.1, trap the agent within the U-shaped obstacle .

Increasing the noise level alleviates this issue (Fig. S1c). Higher stochasticity allows the agent to occasionally escape the U-shaped region; once outside, the repulsive force becomes beneficial again, pushing the agent away from the obstacle and toward the goal. Thus the noise level regulates to what extent goal-directed planning can overcome obstacles by transiently moving away from the goal.

This simple planning method provides an alternative to the navigation strategy of [58]. Note that even more difficult obstacles can be handled with higher dimensional cognitive maps as considered in Section 2.2. But there are certainly also limitations to the problem solving capability of a simple heuristic online algorithm that is equipped with just a single cognitive map. For example, to escape from an U-shaped obstacle one would need higher and higher levels of noise when the U-shape becomes deeper. Then a hierarchical organization becomes more appropriate, where a problem is divided on the top level into subproblems that each require different solution methods. An interesting open problem is whether a more generally approach with several hierarchically structured cognitive maps could achieve that.

B Possible implementation of the generation of cognitive maps by CMLs and GCMLs

The cognitive map is generated for the GCML in the same way as for the CML. The latter was described in detail in [26], and two network diagrams are repeated here for the convenience of the reader.

The architecture consists of several populations of neurons. Peripheral neurons encode observations (blue in Fig. S2), and other neurons encode actions, using one-hot coding (red in Fig. S2).

Delay modules introduce one-time-step delays through inhibitory relays. Such delay modules are readily available in neuromorphic hardware platforms, including Spinnaker and Intel's Loihi chip [105], as well as for in-memory computing systems.

As illustrated in Fig. S2, the model learns the parameters $\mathbf{Q}$, $\mathbf{V}$ through minimizing the prediction error $\mathbf{Qo}_{t+1} - \mathbf{Qo}_T - \mathbf{Va}_t$ (Fig. S2(a)), while $\mathbf{W}$ is updated via Hebbian plasticity (Fig. S2(b)).

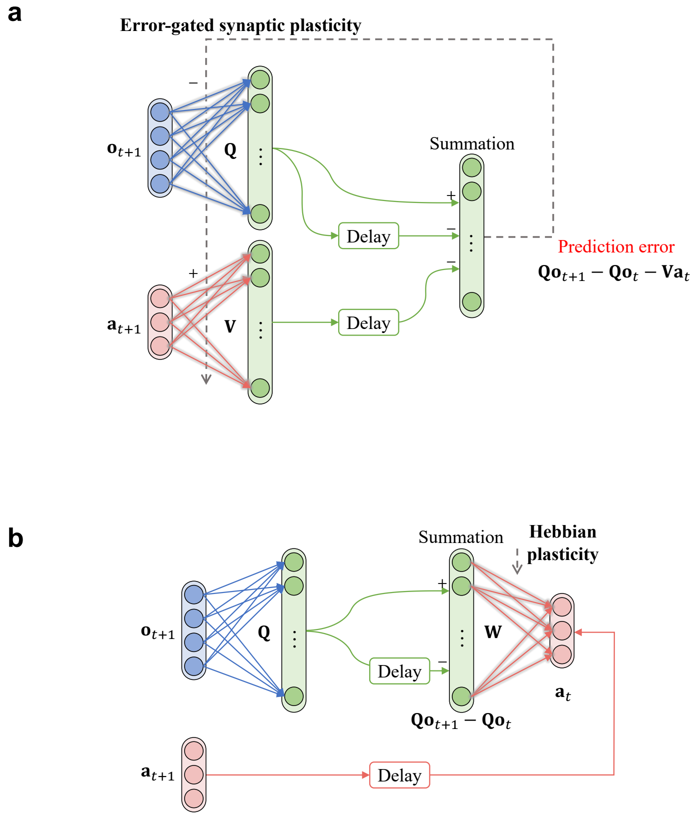

Fig. S2: **Generation of cognitive maps for the CML and GCML through self-supervised learning (copied from [26]). a** Network structure for cognitive map learning. The prediction error, computed by the population of linear units on the right, gates synaptic plasticity between neuron populations representing observations, actions, and internal states of the cognitive map. **b** Network structure for learning the weight matrix $\mathbf{W}$ (inverse model) using Hebbian plasticity. Some signals pass through inhibitory interneurons (not shown), indicated by a negative sign at the corresponding synaptic connections. These signals are assumed to be delayed by one time step.

C Scaling properties of the CML and GCML

We scale up the graph size and average path length, and also the dimension of the state space, in order to see how the performance of the GCML degrades.

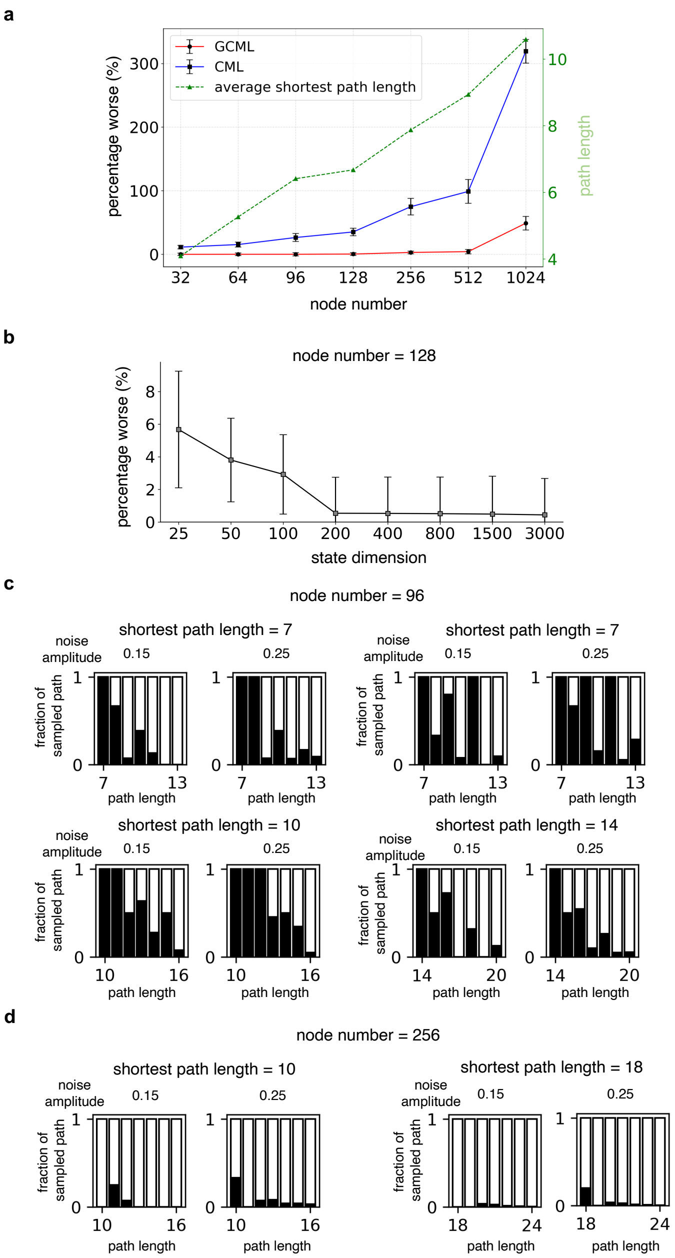

Fig. S3: **Scaling of the GCML performance on random graphs.** (a) Increase of the minimal length of paths produced by the CML and GCML with increasing size of the random graph for random start and goal nodes, in comparison with the shortest path produced by the Dijkstra algorithm. One sees that the performance of the GCML degrades substantially more gracefully. (b) Dependence of the minimal length of paths (again in comparison with the shortest path produced by the Dijkstra algorithm) that are produced by the GCML for a random graph with 128 nodes, shown here as function of the dimension of the state space. One sees that performance improves when the dimension of the state space increases. Mean percentage worse is shown by the center points, and standard deviations are shown by the error bands, computed over 100 random start–goal node pairs in (a) and (b). (c, d) Distribution of the lengths of paths produced by the GCML for random graphs with increasing number of nodes. As in Fig. 3 c,d the length of the black bar indicates for each path length the fraction of paths of that length from the given start to the given goal that are generated by the GCML. Panel c shows results for graphs with 96 nodes, and panel d for graphs with 256 nodes. In comparison with Fig. 3c, d for random graphs with 32 nodes one sees here that a decent approximate solution to the k-shortest path problem is still provided for graphs with 96 nodes, but not for graphs with 256 nodes.

We first examine how the performance of the CML and the GCML degrades when the average shortest-path length increases. The results are shown in Fig. S3a as functions of the number of nodes in the graph. The average shortest-path length between node pairs increases from 2.90 to 7.60 (green curve and y-axis on the right). Performance is quantified as the relative path-length increase over the Dijkstra shortest path:

$$p = \frac{l - l_{\text{Dijkstra}}}{l_{\text{Dijkstra}}}, \tag{32}$$

where l is the path length generated by the CML or GCML, and l_{Dijkstra} is the shortest-path length computed by the Dijkstra algorithm. For each graph size, 100 random start–goal pairs are evaluated.

Note that the performance of the GCML degrades significantly more slowly than that of the CML. The dimension of the high-dimensional state space is set to 3,000 for both models. Exploration is carried out using 2,000 trajectories, each consisting of 128 steps. For the GCML, the number of sampled trajectories is fixed to 40 and the noise scale is fixed to 0.15. All other settings match Section 4.4. To generate random graphs with N nodes, in contrast to Section 4.4, we first constructed a ring structure by connecting nodes sequentially, and then randomly added $N/4$ additional edges between randomly selected node pairs. This process allows to control the sparsity of the graph, while making sure that there exists a path between any pair of nodes. A sparser graph leads to longer average shortest path length between all pairs of nodes in the graph.

Impact of the dimension of the state space on performance. We fixed the number of nodes at 128 and varied the dimensionality of the high-dimensional state space. As shown in Fig. S3b, GCML performance improves as the state dimension increases from 25 to 3,000. All other parameters remain identical to those in the path-length scaling experiment. Performance and standard deviations are computed over 100 random start–goal node pairs.

Scaling of GCML performance in approximating the k shortest-path problem. As described in Section 2.2, the GCML provides an approximate solution to the k shortest-path problem. Here, we evaluate how the quality of these approximate solutions degrades as the number of nodes in the graph increases. The results are shown in Fig. S3c,d. In both the 96-node graphs (Fig. S3c) and the 256-node graphs (Fig. S3d) the dimension of the state space of the GCML is fixed at 3,000, and all other parameters remain unchanged. According to Fig. S3c, the GCML performs well for four randomly selected node pairs in 96-node graphs, with shortest-path lengths ranging from 7 to 14. However, when the number of nodes increases to 256 (Fig. S3d), GCML fails to generate any valid shortest path of length 10 or 18 between the given start and goal nodes when the noise scale is 0.15. Increasing the noise scale to 0.25 alleviates this issue to some extent, but the GCML still does not generate all possible short path lengths.

D Details of the GCML planning algorithm for sections 2.2 and 2.3.

Algorithm 1 provides all details of the GCML algorithm for the abstract graph task, and Algorithm 2 for the silhouette decomposition task. All notations are consistent with those used in the Methods section.

Algorithm 1 Generating Trajectories in the Abstract Graph Task

Require: Initial observation $\mathbf{o}_0$, target observation $\mathbf{o}^*$, number of trajectories n_θ, trajectory length n_{traj}, trained GCML

Compute target state: $\mathbf{s}^* \leftarrow \mathbf{Q}\mathbf{o}^*$
for $\theta = 0$ to $n_\theta - 1$ **do**
    Initialize current state: $\hat{\mathbf{s}}_t \leftarrow \mathbf{Q}\mathbf{o}_0$
    for $t = 0$ to $n_{traj} - 1$ **do**
        if $\hat{\mathbf{s}}_t \approx \mathbf{s}^*$ **then**
            break
        end if
        if $t = 0$ **then**
            Set affordance factor $\hat{\mathbf{g}}$ using real affordance from $\mathbf{o}_0$
        else
            Estimate affordance vector: $\hat{\mathbf{g}} \leftarrow \mathbf{G}\hat{\mathbf{s}}_t$
        end if
        Compute delta vector: $\mathbf{\Delta} \leftarrow \mathbf{s}^* - \hat{\mathbf{s}}_t$
        Compute utility with noise: $\mathbf{u} \leftarrow \mathbf{W}\mathbf{\Delta} + \epsilon$
        Compute affordance factor: $\hat{\mathbf{g}}_t = \mathbf{G}\hat{\mathbf{s}}_t$
        Compute eligibility: $\mathbf{e} \leftarrow \hat{\mathbf{g}}_t \otimes \mathbf{u}$
        Select next action: $\mathbf{a}_t \leftarrow \mathrm{WTA}(\mathbf{e})$
        Predict next state: $\hat{\mathbf{s}}_{t+1} \leftarrow \hat{\mathbf{s}}_t + \mathbf{V}\mathbf{a}_t$
    end for
end for
return a trajectory from the given start to the given goal.

Algorithm 2 Generating a Set of Possible Decompositions in the Silhouette Task

Require: Initial silhouette $\mathbf{o}_0$, target observation $\mathbf{o}^* = \mathbf{0}$, maximum trajectory length n_{traj}, trained cognitive map

Initialize state: $\hat{\mathbf{s}}_0 \leftarrow \mathbf{Q}\mathbf{o}_0$
for $t = 0$ to $n_{traj} - 1$ **do**
    if $\hat{\mathbf{s}}_t \approx \mathbf{s}^*$ **then**
        Found a valid decomposition for $\mathbf{o}_0$
        break
    end if
    Compute delta vector: $\mathbf{\Delta} \leftarrow \mathbf{Q}\mathbf{o}^* - \hat{\mathbf{s}}_t$
    Apply context-dependent affordance factor g_2 (see Equ. 23):
        $\mathrm{g}_2(\mathbf{\Delta}) = (\mathbf{k} * (\mathbf{1} + \mathbf{\Delta})) \otimes (-\mathbf{\Delta})$
    if no valid action remains **then**
        No valid decomposition found.
        break
    end if
    Compute eligibility with noise: $\mathbf{e}_t \leftarrow \mathrm{g}_1(\mathbf{W}\mathrm{g}_2(\mathbf{\Delta})) + \epsilon$ (see Equ. 24)
    Select action: $\mathbf{a}_t \leftarrow \mathrm{WTA}(\mathbf{e}_t)$
    Compute next state: $\hat{\mathbf{s}}_{t+1} = \hat{\mathbf{s}}_t + \mathbf{V}\mathbf{a}_t$
end for
return a generated decomposition plan

E Cognitive maps for two further instances of the tiling-decomposition task considered in Section 2.3.

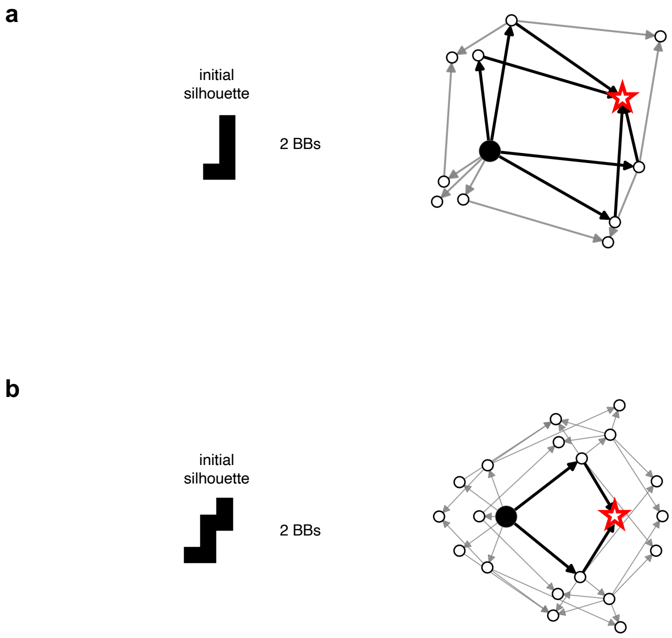

Fig. S4: **Two further examples for the cognitive map that the GCML generates for the tiling problem.** Panels (a) and (b) show the cognitive map of a silhouette containing two BBs. The left panels display the initial silhouette, and the right panels present 2D projections of their corresponding cognitive maps. The black node represents the high-dimensional state $\mathbf{s}_0$ of the initial silhouette, and the red star denotes the high-dimensional state $\mathbf{s}^*$ of the empty goal silhouette. Each arrow corresponds to the embedding of an action (i.e., removing a BB from a silhouette). All nodes are calculated following Eq. 23. All 2-D projections are generated via t-SNE (PCA initialized). The solid black arrows indicate trajectories that successfully decompose the initial silhouettes.

Fig.S4 shows 2D t-SNE projections for two further instances of the compositional task addressed in Fig.4. One sees that also for these examples the cognitive maps provide a sense-of-direction for decomposing the given silhouette.
